# Canadian Hematology Consensus Group Recommendations for the Management of Relapsed and/or Refractory Follicular Lymphoma

**DOI:** 10.3390/curroncol33080451

**Published:** 2026-07-28

**Authors:** Carolyn Owen, Christopher Lemieux, Mark Bosch, Kelly Davison, Nicholas Forward, Roopesh Kanasara, Mary Margaret Keating, Anca Prica, Colin Stewart, Abi Vijenthira, Laurie H. Sehn

**Affiliations:** 1 Division of Hematology and Hematological Malignancies, University of Calgary, Calgary, AB T2N 1N4, Canada; 2 Division of Hematology and Medical Oncology, Centre Intégré de Cancérologie, CHU de Québec-Université Laval, Québec, QC G1J 0J9, Canada; 3 Hematological Oncology, University of Saskatchewan, Saskatoon, SK S7N 5A2, Canada; 4 Department of Medicine, McGill University, Montreal, QC H3A 0G4, Canada; 5 Division of Hematology, Queen Elizabeth II Health Sciences Centre, Dalhousie University, Halifax, NS B3H 4R2, Canada; 6 Department of Internal Medicine, Cancer Care Manitoba, University of Manitoba, Winnipeg, MB R3E OV9, Canada; 7 Division of Medical Oncology and Hematology, Princess Margaret Cancer Centre, Toronto, ON M5G 2C4, Canada; 8 BC Cancer Centre for Lymphoid Cancer, The University of British Columbia, Vancouver, BC V6T 1Z4, Canada

**Keywords:** hematology, follicular lymphoma, oncology, guidelines, Canada

## Abstract

Relapsed and/or refractory follicular lymphoma (R/R FL) remains a therapeutic challenge due to its chronic relapsing course and increasingly complex treatment landscape. Novel therapies, including immunomodulatory combinations, bispecific antibodies (BsAbs), Bruton tyrosine kinase inhibitors, and chimeric antigen receptor (CAR) T-cell therapies, have expanded treatment options and increased the complexity of treatment selection and sequencing. The Canadian Hematology Consensus Group (CHCG) convened a national panel of lymphoma experts to develop evidence-informed consensus recommendations for the management of adults with R/R FL in the Canadian context. Clinical questions informed a structured literature review of studies published through February 2026, including randomized trials, phase II studies, observational data, conference proceedings, and relevant guidelines. Recommendations were developed using a modified Delphi consensus process and graded using a framework adapted from the British Committee for Standards in Haematology. Key recommendations include repeat biopsy to exclude histologic transformation at relapse, individualized treatment selection based on timing of relapse and patient-specific factors, preferential use of lenalidomide-rituximab (LenR)-based triplet combinations in most second-line settings, and incorporation of BsAb and CAR T-cell therapy in third-line and later disease. These recommendations aim to provide practical guidance for Canadian clinicians managing patients with R/R FL.

## Simple Summary

There have been many advances in therapeutic options for relapsed/refractory follicular lymphoma (R/R FL) in Canada and internationally. Herein, we provide a summary of the landmark studies leading to approval of different therapies and consensus recommendations for the management of R/R FL in Canada.

## 1. Introduction

Non-Hodgkin lymphoma (NHL) is the most common hematologic malignancy in Canadian adults and is projected to account for approximately 5.2% of all new cancer diagnoses in men and 4.2% in women in 2025 [1,2]. According to Canadian Cancer Statistics, an estimated 12,000 new cases of NHL will be diagnosed in Canada in 2025 [1,2]. Follicular lymphoma (FL) is the most common indolent B-cell lymphoma and represents approximately 20–35% of all NHL cases in North America [1].

First-line therapy for FL typically consists of chemoimmunotherapy combining cytotoxic chemotherapy with an anti-CD20 monoclonal antibody (MAb), which has been shown to produce high response rates and prolonged progression-free survival (PFS) [3,4,5,6,7,8]. For patients responding to frontline chemoimmunotherapy, maintenance rituximab was also shown to provide a clinically meaningful improvement in PFS, but without an OS improvement. Rituximab monotherapy is thus commonly used to prolong the first remission but may be omitted for individual patients based on risk factors/patient choice. Despite the effectiveness of frontline treatment, FL remains characterized by a chronic relapsing course, and most patients will experience disease recurrence and require subsequent lines of therapy [2,9].

The management of relapsed and/or refractory (R/R) FL has evolved substantially over the past decade with the introduction of novel targeted therapies, immunotherapies, and cellular therapies, resulting in an increasingly complex treatment landscape. Several therapeutic options are now available beyond conventional immunochemotherapy, including immunomodulatory combinations, Bruton tyrosine kinase (BTK) inhibitors, bispecific antibodies (BsAbs), and chimeric antigen receptor (CAR) T-cell therapy. As new data and regulatory decisions continue to emerge, Canadian hematologists must navigate treatment selection while considering prior therapy, disease biology, comorbidities, treatment goals, and provincial access.

The Canadian Hematology Consensus Group (CHCG) was established by Canadian lymphoma specialists to develop timely national consensus recommendations in hematologic malignancies. The CHCG initiative aims to synthesize current evidence and expert clinical experience to provide practical guidance for Canadian clinicians.

In this paper, members of the CHCG together with a panel of lymphoma experts from across Canada present national consensus recommendations for the management of patients with R/R FL. The goal of this document is to provide evidence-informed guidance to support clinicians in treatment selection and sequencing of therapies in the contemporary Canadian clinical context. These recommendations are not intended to replace clinician judgement for individual cases. This paper has been endorsed by the Leukemia & Lymphoma Society of Canada (LLSC).

## 2. Methods

### 2.1. Scope and Target Population

These recommendations apply to patients with FL who have relapsed following prior systemic therapy. Patients previously treated with radiotherapy alone for limited-stage disease who subsequently require systemic treatment should generally be approached according to first-line FL treatment recommendations. Treatment for patients with FL who have histologic transformation to DLBCL, as confirmed by tissue biopsy, should follow DLBCL guidelines, which are not described in this consensus document.

### 2.2. Target Users

These recommendations are intended for physicians involved in the care of patients with FL. The recommendations are designed to support clinical decision-making regarding treatment selection, sequencing of therapies, and the evaluation of patients in routine clinical practice.

### 2.3. Panel Composition and Development of Clinical Questions

A national panel of eleven hematologists with expertise in lymphoma was convened to develop this consensus statement. Panel members represented multiple regions across Canada, providing broad geographic and practice-setting representation. Panel members were asked to identify key clinical questions relevant to the management of patients with R/R FL. These questions were refined through discussion to focus on areas of greatest clinical value. The final set of clinical questions was used to guide the literature search and the development of consensus recommendations. The manuscript is organized by clinical question, with each section including a summary of the relevant evidence, consensus recommendations, and the corresponding level of evidence.

### 2.4. Literature Search Strategy

A literature review was conducted to identify studies relevant to the clinical questions addressing the management of R/R FL. Search strategies were developed individually for each clinical question based on the relevant clinical concepts and therapeutic interventions being evaluated. Medical Subject Headings (MeSH) terms and keywords were used as appropriate for each topic. Screening of titles, abstracts, and full-text articles and extraction of data from eligible studies into evidence tables were performed by a medical writer in collaboration with the lead authors.

MEDLINE databases and abstracts from major international conferences, including the American Society of Hematology (ASH), the American Society of Clinical Oncology (ASCO), and the European Hematology Association (EHA), were searched through 28 February 2026. The specific search terms and strategies used for each clinical question are summarized in the Supplementary Materials.

Eligibility criteria were defined according to the clinical question being addressed, but generally included randomized controlled trials (RCTs), phase II trials, prospective or retrospective cohort studies, case series, systematic reviews, and relevant clinical practice guidelines involving adult patients with FL. Studies were limited to those published in English. Conference abstracts were included when relevant clinical data were available. Lower quality data was not summarized when randomised clinical trial evidence was available. For clinical questions focusing on treatment selection, only studies that investigated therapies that are currently approved or have been submitted for approval by Health Canada were included.

### 2.5. Consensus Process

Following completion of the literature review and development of evidence summaries, panel members reviewed available evidence for each clinical question. Evidence tables summarizing the key studies and clinical outcomes were circulated to all panel members in advance of the consensus discussions.

Consensus on the proposed statements was reached using a modified Delphi process [10]. All drafted recommendations were included in a web-based survey where all panelists responded. Panelists rated agreement with each statement using a 5-point scale and voted on whether it should be included in the final manuscript. Free-text feedback was collected for areas of disagreement. Panel members considered the strength and consistency of the evidence, the clinical relevance of available data, and the applicability of findings to the Canadian healthcare setting, including considerations related to treatment access and reimbursement. Statements with minor or major rewording were reviewed by lead authors and those with major rewording were included in a second-round survey. Consensus was defined as ≥80% agreement among panel members. To assess the level of consensus among a broader group of Canadian FL experts, the final recommendations were also circulated to an additional group of hematologists with expertise in FL. Consensus was maintained, with >80% agreement for all recommendations when the results from the original expert panel and the broader reviewer group were combined.

Lead authors graded the recommendations based on the strength of the evidence. Panel members reviewed and approved the final recommendations during a final consensus meeting.

### 2.6. Grading of Evidence and Recommendations

The strength of recommendations and quality of evidence supporting each recommendation were graded using a framework adapted from the British Committee for Standards in Haematology (BCSH) (Table 1) [11]. For each clinical question, the panel reviewed the available evidence and formulated consensus recommendations. The assigned strength of recommendation and level of evidence are reported alongside each recommendation statement.

## 3. Consensus Recommendations

**Figure 1:**
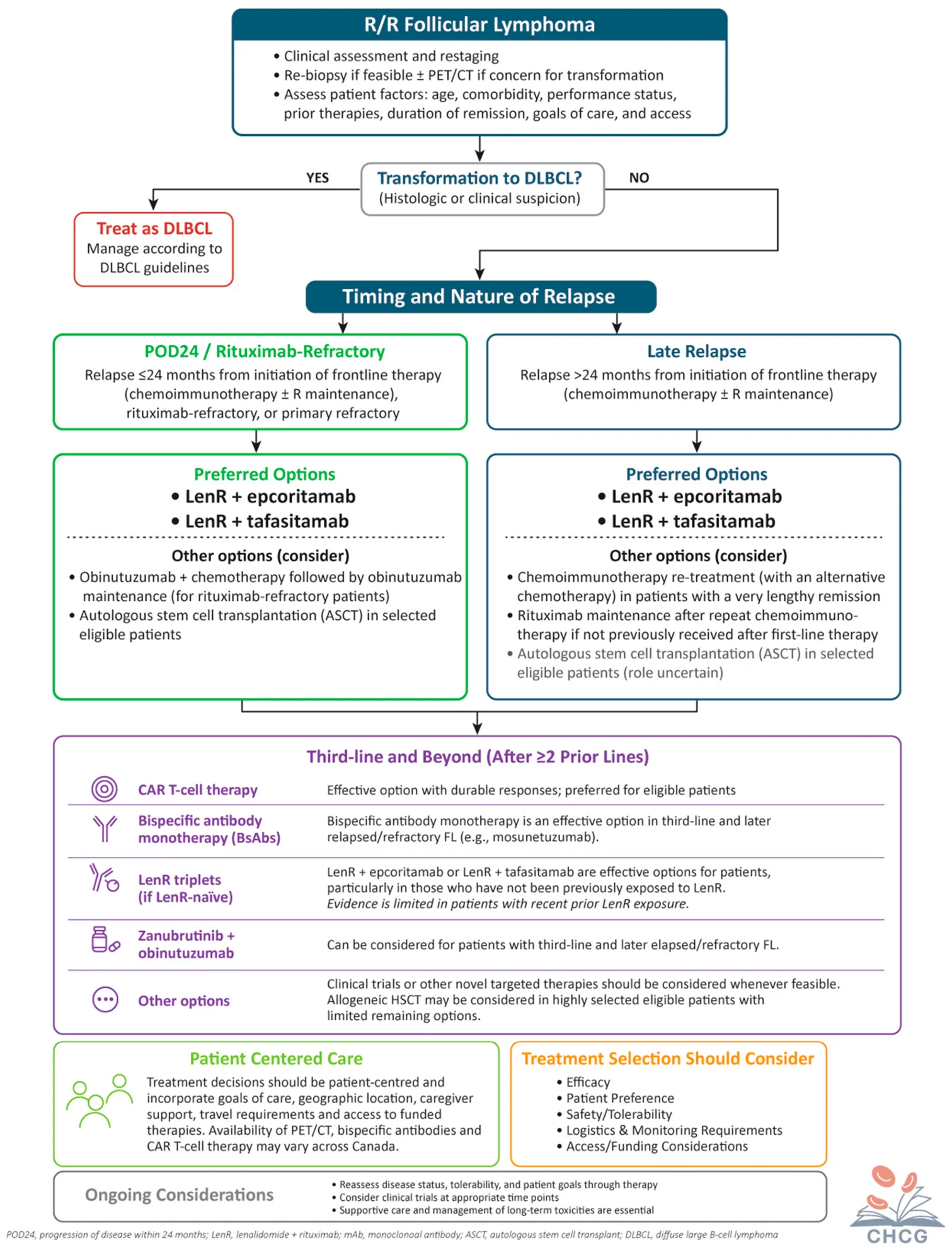
CHCG Treatment Algorithm for Relapsed/Refractory Follicular Lymphoma (R/R FL).

A summary of the consensus recommendations is provided in Table 2, and a treatment algorithm is shown in Figure 1. These recommendations achieved >80% consensus in the original 11-member expert panel. The 80% concordance was maintained in the 16-member external panel, except for the management of POD24, where there remains debate on the role of ASCT.

### 3.1. Indications for Treatment

**Clinical Question 1.** When is therapy indicated for relapsed/refractory follicular lymphoma (FL)?

**Recommendation 1.** Asymptomatic patients with relapsed FL may be managed with active surveillance, with therapy deferred until symptomatic disease or progressive disease meeting GELF criteria.

**Consensus achieved:** 100% agreement (91% strongly agree, 9% agree); 100% of panelists supported inclusion of this recommendation.

**Strength of recommendation:** Grade 1B

**Recommendation 2.** Treatment for relapsed/refractory follicular lymphoma is recommended for any patient with symptomatic lymphoma.

**Consensus achieved:** 100% agreement (91% strongly agree, 9% agree); 100% of panelists supported inclusion of this recommendation.

**Strength of recommendation:** Grade 1A

**Rationale**

In general, indications for treatment in the relapsed setting are similar to those used in the first-line management of FL and are reflected in Canadian provincial and international guidelines [5,12,13,14,15,16]. Commonly used criteria include the Groupe d’Étude des Lymphomes Folliculaires (GELF) [17] and the British National Lymphoma Investigation (BNLI) [18], both of which were developed based on studies evaluating immediate treatment versus active surveillance in previously untreated FL.

No prospective studies have directly compared immediate treatment versus observation in patients with asymptomatic, low tumour burden FL at relapse following prior anti-lymphoma therapy. Nevertheless, existing guidelines generally support observation for selected patients with low tumour burden relapsed disease, with these recommendations largely extrapolated from studies conducted in the first-line setting [5,12,13,14,15,16].

More recent studies in previously untreated low tumour burden FL suggest that early rituximab monotherapy may delay the need for subsequent treatment without improving OS [19]. However, comparable prospective data in the relapsed setting are lacking. Consequently, active surveillance remains an appropriate management strategy for selected patients with asymptomatic, low tumour burden relapsed FL, supported by retrospective series demonstrating favorable outcomes with observation in this population [20].

### 3.2. Baseline Evaluation Prior to Treatment

**Clinical Question 2.** When should positron emission tomography (PET) scans be considered?

**Recommendation 1.** A PET scan should be considered in patients with concern for histologic transformation.

**Consensus achieved:** 90% agreement (50% strongly agree, 40% agree); 100% of panelists supported inclusion of this recommendation.

**Strength of recommendation:** Grade 2B

**Additional** Considerations

Positron emission tomography/computed tomography (PET/CT) should be considered in patients with clinical, biochemical, or radiologic features suggestive of histologic transformation, particularly to identify the most FDG-avid site to guide biopsy.

PET/CT may also provide a useful baseline when functional imaging is anticipated for subsequent response assessment.

**Rationale**

Contrast-enhanced computed tomography (CT) or positron emission tomography/computed tomography (PET/CT), where available, should be performed for disease restaging when relapse is suspected based on clinical or laboratory findings.

International guidelines support cross-sectional imaging for lymphoma staging and response assessment [3,4,5]. While PET/CT is preferred for initial staging, evidence supporting its routine use in the relapsed setting is more limited. In the Canadian context, access to PET/CT varies across provinces, and contrast-enhanced CT remains a widely available and appropriate modality for disease restaging.

Histologic transformation of FL to an aggressive lymphoma, as confirmed by tissue biopsy, is associated with a poorer prognosis and requires prompt diagnosis and treatment [21,22]. Identification of histologic transformation can be challenging based on clinical and radiologic features alone [3,4,5]. Observational studies have demonstrated that higher maximum standardized uptake values (SUVmax) on PET imaging are associated with histologic transformation compared with indolent FL [23,24] (Supplementary Table S2). However, these data are limited by small sample sizes, heterogeneity in study populations, and inconsistent SUVmax thresholds. Importantly, imaging alone cannot reliably distinguish histologically transformed disease from indolent disease [3,4,5].

Biopsy remains the gold standard for confirming histologic transformation [3,4,5]. International guidelines, including those from the ESMO, NCCN, and Lugano, support the use of PET/CT to assist in identifying appropriate biopsy sites in patients with suspected transformation.

### 3.3. Role of Re-Biopsy

**Clinical Question 3.** When should re-biopsy be performed?

**Recommendation 1.** Re-biopsy should be considered if feasible in all patients with relapsed/refractory FL to confirm relapse and exclude histologic transformation.

**Consensus achieved:** 100% agreement (90% strongly agree, 10% agree); all panelists supported inclusion of this recommendation.

**Strength of recommendation:** Grade 1B

**Rationale**

Histologic transformation of FL to an aggressive lymphoma, most commonly DLBCL, is a clinically significant event associated with inferior outcomes compared with relapsed indolent disease [22]. Reported rates of histologic transformation have historically been estimated at approximately 2–3% per year [21,22]; however, more recent studies suggest lower cumulative rates of approximately 5–8% at 5 years [25,26,27].

Several clinical factors have been associated with an increased risk of transformation, including elevated LDH [21], advanced-stage disease [22], higher-grade histology [28], early disease progression (POD24) [29], anemia [28], impaired performance status [28], and the presence of B symptoms [28]. Given the heterogeneity in clinical presentation, histologic transformation cannot be reliably identified based on clinical or radiologic features alone.

Outcomes for patients with histologic transformation are consistently worse than for those with relapsed indolent FL. In a Canadian cohort, patients who developed histologic transformation had a 10-year overall survival (OS) of 36% compared with 75% among those without transformation [22]. Similar findings have been observed in other cohorts, with median OS following histologic transformation of approximately 3–5 years [21,28].

Despite the lack of high-level prospective data evaluating the role of routine re-biopsy, confirmation of histologic transformation directly impacts treatment selection and international guidelines recommend biopsy to confirm transformation in patients with suspected relapsed disease [3,4,5]. Re-biopsy may also provide clinically relevant information regarding antigen expression (e.g., CD20, CD19), which may inform treatment selection, including targeted therapies and cellular therapies.

Historically, excisional biopsy has been the gold standard for lymphoma; however, it is often not feasible or timely [30]. Image-guided core needle biopsies provide a less invasive and timely alternative, particularly for deep or difficult-to-access nodes. Recent improvements in radiological techniques have shown that image-guided core needle biopsies often provide sufficient tissue for diagnosis, with comparative evidence suggesting fewer AEs than surgical biopsy [30,31,32].

### 3.4. Management of Early Relapse (POD24)

**Clinical Question 4.** What is the optimal second-line therapy for FL patients who relapse within 2 years of chemoimmunotherapy +/− R-maintenance (POD24) or who are R-refractory?

**Recommendation 1.** Patients who experience early recurrence of FL (POD24) should have a biopsy to exclude histologic transformation; patients with histologic transformation should be treated as per DLBCL recommendations.

**Consensus achieved:** 100% agreement (91% strongly agree, 9% agree); all panelists supported inclusion of this recommendation.

**Strength of recommendation:** Grade 1A

**Rationale**

Progression of disease within 24 months of initiation of frontline therapy (POD24) is associated with inferior survival outcomes and an increased risk of histological transformation [22]. Histologic transformation most commonly occurs in DLBCL and has historically been reported at an incidence of approximately 2–3% per year in patients with FL [21,22]. Patients with POD24 appear to have a particularly high risk of transformation and adverse clinical outcomes compared with patients experiencing later relapse [29,33,34,35].

Given the therapeutic and prognostic implications of transformation, repeat biopsy at the time of early relapse is strongly recommended whenever feasible to confirm histology and exclude transformed disease. Clinical features that may increase suspicion for histologic transformation include rapidly progressive lymphadenopathy, new B symptoms, rising lactate dehydrogenase, extranodal disease, or discordant PET findings; however, histologic transformation cannot be reliably excluded on clinical grounds alone [21,22,28,29].

This consensus paper does not specifically address the management of histologically transformed FL. Consistent with international guidelines, patients with biopsy-confirmed transformation should generally be managed according to treatment recommendations for DLBCL [5,12,13,14,15,16].

**Recommendation 2.** Lenalidomide-rituximab (LenR) triplets (LenR + epcoritamab and LenR + tafasitamab) are recommended therapy for patients with early relapse of FL.

**Consensus achieved:** 91% agreement (27% strongly agree, 64% agree; 9% disagree); 82% of panelists supported inclusion of this recommendation.

**Strength of recommendation:** Grade 1B

**Rationale**

Patients with POD24 represent a high-risk subgroup of FL associated with inferior survival outcomes compared with patients experiencing later relapse [36]. In the AUGMENT study, approximately one-third of patients had POD24; however, dedicated efficacy analyses for the POD24 subgroup were not reported [37]. Nevertheless, AUGMENT established LenR as an effective treatment regimen in relapsed FL, demonstrating improved PFS compared with rituximab-placebo in the overall FL population (median PFS 39.4 vs. 13.9 months; HR 0.40, *p* < 0.0001) (Supplementary Tables S3 and S4).

Recent studies evaluating the addition of novel antibody-based therapies to the LenR backbone have demonstrated clinically meaningful improvements in efficacy outcomes, compared with LenR-based control arms, including in patients with POD24 (Supplementary Tables S3 and S4). In the phase III inMIND study, tafasitamab-LenR improved PFS in the overall study population compared with placebo-LenR (median PFS 22.4 vs. 13.9 months; HR 0.43, 95% CI 0.32–0.58), with improvements also observed in ORR (84% vs. 72%) and CR rate (45% vs. 34%; OR 2.0, *p* = 0.0014) [38]. Approximately one-third of enrolled patients had POD24 (31–32%), and in this subgroup, median PFS was 19.2 months with tafasitamab-LenR versus 11.3 months with placebo-LenR (HR 0.43, 95% CI 0.27–0.69). Benefit was also maintained in patients refractory to prior anti-CD20 therapy (HR 0.44, 95% CI 0.30–0.65).

Similarly, the phase III EPCORE FL-1 study demonstrated substantial improvements with epcoritamab-LenR compared with LenR alone [39]. Approximately 60% of patients enrolled had POD24. In the overall study population, median PFS was not reached with epcoritamab-LenR versus 11.7 months with LenR alone (HR 0.21, 95% CI 0.14–0.31), with ORR of 95% versus 75% and CR rates of 83% versus 50%, respectively. In patients with POD24 specifically, CR rates were 80.3% versus 38.7%, while median PFS was not reached versus 9.6 months (HR 0.22, 95% CI 0.13–0.37). Similar benefit was observed in patients with double-refractory disease, where median PFS was 21.9 versus 10.9 months (HR 0.23, 95% CI 0.13–0.39).

Although no direct comparative studies between LenR + epcoritamab and LenR + tafasitamab are available, cross-trial comparisons suggest potential differences in efficacy, toxicity, and treatment logistics. Epcoritamab-LenR may produce deeper and more durable responses, with higher CR rates observed in cross-trial comparisons, including in patients with POD24 (CR rate 80.3% in EPCORE FL-1) and median PFS not reached in both the overall and POD24 populations [38,39]. However, differences in study populations, proportion of high-risk patients, and trial design limit definitive efficacy comparisons between studies. Tafasitamab-LenR may offer a more favorable tolerability and operational profile, as it is not associated with cytokine release syndrome (CRS) or immune effector cell-associated neurotoxicity syndrome (ICANS), and does not require step-up dosing, intensive monitoring, or hospitalization during treatment initiation. In contrast, epcoritamab-LenR was associated with CRS in 35% of patients (all grade 1/2), use of tocilizumab in 25%, grade 3/4 infections in 33%, and treatment discontinuation in 19% of patients, compared with discontinuation rates of 9% with tafasitamab-LenR in the inMIND study.

**Recommendation 3.** If chemoimmunotherapy is used for rituximab-refractory FL patients, obinutuzumab is the preferred monoclonal antibody.

**Consensus achieved:** 100% agreement (64% strongly agree, 36% agree); all panelists supported inclusion of this recommendation.

**Strength of recommendation:** Grade 1B

**Rationale**

Chemoimmunotherapy remains an evidence-based option for selected patients with relapsed/refractory FL, particularly when novel therapies are not available, not appropriate, or when a time-limited treatment approach is preferred. The strongest randomized evidence in rituximab-refractory disease comes from the phase III GADOLIN study, which evaluated bendamustine-obinutuzumab followed by obinutuzumab maintenance versus bendamustine alone [40] (Supplementary Tables S3 and S4). However, this study was conducted before the era of targeted therapies and in the current treatment landscape, it is generally not preferred when effective novel-agent combinations are available.

In GADOLIN, bendamustine-obinutuzumab significantly improved PFS compared with bendamustine alone in the overall study population, with a median PFS of 25.8 versus 14.1 months (HR 0.57; 95% CI, 0.44–0.73; *p* < 0.001). In the FL population, median PFS was 25.3 versus 14.0 months (HR 0.52; 95% CI, 0.39–0.69; *p* < 0.001), and an OS benefit was also observed, with median OS not reached versus 53.9 months (HR 0.58; 95% CI, 0.39–0.86; *p* = 0.006). Benefit was maintained in refractory subgroups, including double-refractory patients, where 1-year PFS was 67% versus 51% and the PFS HR was 0.56, as well as rituximab mono-refractory patients, where 1-year PFS was 78% versus 68% and the PFS HR was 0.37.

However, the applicability of GADOLIN to the POD24 setting is limited because POD24 status was not reported [40]. The trial population was enriched for refractory disease, with all patients rituximab-refractory and approximately 79–80% of patients in the FL population double-refractory, but it does not directly define outcomes for patients with early relapse after frontline BR with or without rituximab maintenance. Additionally, it included patients who had not received bendamustine and rituximab followed by rituximab maintenance (the current Canadian first-line standard of care), reducing the generalisability of this study for Canadian patients today.

Treatment selection should also consider toxicity. Bendamustine-obinutuzumab was associated with substantial hematologic toxicity and infections, including grade 3/4 neutropenia in 34.8% versus 27.1%, febrile neutropenia in 5.9% versus 3.4%, grade 3/4 infections in 22.5% versus 19.2%, and treatment discontinuation due to AEs in 20% versus 17% compared with bendamustine alone [40]. Given the infection and marrow toxicity associated with bendamustine-based therapy, repeat bendamustine-containing chemoimmunotherapy is unappealing for patients who relapse early after frontline BR, particularly those with short remission duration, persistent cytopenias, recurrent infections, or cumulative treatment-related toxicity.

**Recommendation 4.** Autologous stem cell transplantation can be considered for younger, fit patients with POD24 R/R FL.

**Consensus achieved:** 91% agreement (9% strongly agree, 64% agree; 18% neutral; 9% disagree); all panelists supported inclusion of this recommendation.

**Strength of recommendation:** Grade 2B

**Rationale**

ASCT has historically been considered for fit patients with chemosensitive relapsed FL, particularly after early treatment failure; however, supporting evidence is mixed and must be interpreted in the context of evolving frontline therapy and effective non-transplant options.

Randomized data are limited (Supplementary Tables S3 and S4). In the FLAZ12 study, which enrolled a high-risk population in which 90% of patients had received only one prior line of therapy and 33% had POD24, ASCT did not improve outcomes compared with radioimmunotherapy consolidation. At a median follow-up of 77 months, 3-year PFS was 62% in both arms, and 6-year PFS was 46% with ASCT versus 52% with radioimmunotherapy (HR 1.11; 95% CI, 0.69–1.80; *p* = 0.6662). Overall survival was also similar, with 3-year OS of 87% versus 86% and 6-year OS of 76% versus 78%, respectively (HR 0.94; 95% CI, 0.45–1.94; *p* = 0.8588). The study was closed early due to poor accrual, limiting its power to detect differences between groups.

Older randomized data suggested a benefit for high-dose therapy with ASCT compared with chemotherapy alone. In the Schouten study, high-dose therapy with ASCT improved PFS versus chemotherapy (HR 0.30; 95% CI, 0.15–0.61; *p* = 0.0009) and OS (HR 0.40; 95% CI, 0.18–0.89; *p* = 0.026). However, this study was conducted before contemporary rituximab-based treatment approaches and included substantial procedure-related toxicity, with 5 deaths attributed to transplant-related toxicity.

Retrospective analyses provide more supportive evidence for ASCT in selected patients with early treatment failure. In the CIBMTR/NLCS analysis, 5-year OS was numerically higher with ASCT than non-ASCT therapy overall (67% vs. 60%; *p* = 0.16), and the benefit appeared greater among patients receiving ASCT within 1 year of early treatment failure (5-year OS 73% vs. 60%; *p* = 0.05). Similarly, in the GLSG analysis, ASCT was associated with improved 5-year second-line PFS compared with no ASCT (52% vs. 27%; HR 0.47; 95% CI, 0.31–0.70; *p* = 0.00012) and improved 5-year OS (78% vs. 60%; HR 0.53; 95% CI, 0.32–0.86; *p* = 0.0086). In patients with POD24 specifically, ASCT was associated with improved 5-year second-line PFS (51% vs. 19%; HR 0.38; 95% CI, 0.24–0.62; *p* < 0.0001) and OS (77% vs. 59%; HR 0.54; 95% CI, 0.30–0.95; *p* = 0.031).

Despite these data, interpretation is limited by selection bias, differences in transplant eligibility, requirement for chemosensitive disease, and the fact that several studies were conducted before bendamustine-based frontline therapy and newer immunotherapy options. ASCT is also associated with clinically relevant acute and late toxicities. In FLAZ12, grade 3/4 hematologic adverse events during consolidation occurred in 93% with ASCT versus 46% with radioimmunotherapy, and grade 3/4 non-hematologic adverse events occurred in 37% versus 5%. Secondary primary malignancies were reported in 10% versus 4%, respectively.

Overall, ASCT may remain an option for carefully selected, transplant-eligible patients with early relapsed FL who have chemosensitive disease and limited access to preferred novel therapies. However, given the absence of a clear randomized benefit in contemporary studies, the toxicity burden of ASCT, and the availability of active novel regimens, the role of ASCT should be individualized rather than routinely recommended in the second-line setting.

### 3.5. Management of Late Relapse (>24 Months)

**Clinical Question 5.** What is the optimal second-line therapy for FL patients who relapse more than 2 years following initiation of chemoimmunotherapy +/− R-maintenance?

**Recommendation 1.** Patients who experience a late relapse of FL after prior chemoimmunotherapy have a number of possible therapy options. Decisions should be individualized based on patient factors.

**Consensus achieved:** 100% agreement (64% strongly agree, 36% agree); all panelists supported inclusion of this recommendation.

**Strength of recommendation:** Grade 1A

**Rationale**

Patients experiencing late relapse of FL following frontline chemoimmunotherapy ± rituximab maintenance generally have a more favorable prognosis than patients with POD24 and may be appropriate candidates for multiple effective second-line treatment approaches [36]. Available options include LenR-based triplet therapies incorporating CD19-targeted antibodies (e.g., tafasitamab) or CD20-targeted bispecific antibodies (e.g., epcoritamab), repeat chemoimmunotherapy, and ASCT in selected patients (Supplementary Tables S3 and S4) [5,12,13,14,15,16].

There are no randomized trials directly comparing these approaches in patients with late relapse after frontline chemoimmunotherapy ± rituximab maintenance. As a result, no single treatment strategy can be considered optimal for all patients in this setting. Treatment selection should be individualized based on disease tempo and tumour burden, duration and quality of response to prior therapy, prior treatment tolerability, patient age, comorbidities, frailty, patient preferences, logistical considerations, and local access to therapies.

**Recommendation 2.** Lenalidomide-rituximab (LenR) triplet combinations (LenR + epcoritamab or LenR + tafasitamab) are preferred over LenR.

**Consensus achieved:** 100% agreement (73% strongly agree, 27% agree); all panelists supported inclusion of this recommendation.

**Strength of recommendation:** Grade 1A

**Rationale**

As outlined above in the POD24 setting, tafasitamab-LenR and epcoritamab-LenR demonstrated clinically meaningful improvements in PFS and response outcomes compared with LenR-based control arms in broad populations of patients with relapsed FL [38,39].

No direct comparisons between LenR + epcoritamab and LenR + tafasitamab are available. Cross-trial comparisons suggest potential differences in efficacy, toxicity, and logistical complexity, with epcoritamab-LenR potentially associated with deeper and more durable responses, and tafasitamab-LenR potentially associated with a more favorable tolerability and operational profile [38,39]. Treatment selection should therefore be individualized based on patient comorbidities, disease characteristics, logistical considerations, and access to therapy.

**Recommendation 3.** Chemoimmunotherapy re-treatment (with an alternate chemotherapy) may be considered in patients who have experienced a very lengthy remission, but is not a preferred approach assuming the availability of newer combinations incorporating novel agents.

**Consensus achieved:** 91% agreement (18% strongly agree, 55% agree, 18% neutral, 9% disagree); all panelists supported inclusion of this recommendation.

**Strength of recommendation:** Grade 2A

**Rationale**

Chemoimmunotherapy re-treatment may be considered for selected patients with late relapsed FL, particularly those who experienced a very prolonged remission after frontline therapy and remain fit for cytotoxic treatment. In general, a prolonged remission may be considered as a remission lasting approximately one year longer than the expected median PFS achieved with the initial chemoimmunotherapy regimen. For example, following BR, this would correspond to approximately 7 years based on the median PFS of about 6 years reported in the StiL NHL1 study [41]. However, this threshold should not be considered absolute. In clinical practice, re-treatment with chemoimmunotherapy may be appropriate after remissions of approximately 5–10 years, depending on the patient’s age, life expectancy, comorbidities, prior treatment tolerance, and the anticipated need to preserve future treatment options. In the current treatment landscape, re-treatment with chemoimmunotherapy is generally not preferred when effective novel-agent combinations are available [5,12,13,14,15,16].

Evidence supporting chemoimmunotherapy re-treatment in relapsed FL is limited and largely predates current frontline standards (Supplementary Tables S3 and S4). In the phase III study by Rummel et al., BR was compared with fludarabine-rituximab (FR) in patients with relapsed indolent NHL [42]. In the FL subgroup, BR was associated with longer PFS than FR (median 54.5 vs. 22.9 months; HR 0.56, 95% CI 0.34–0.87). In the total study population, BR improved ORR (82% vs. 51%; *p* < 0.0001), CR rate (40% vs. 17%; *p* = 0.0002), and OS (median 109.7 vs. 49.1 months; HR 0.64, 95% CI 0.45–0.91; *p* = 0.012).

The relevance of these data to contemporary practice is limited. Few patients had prior bendamustine exposure; patients refractory to rituximab, bendamustine, or purine analogue therapy were excluded; and outcomes were not reported specifically for patients relapsing >24 months after chemoimmunotherapy [42]. Therefore, these results support the activity of chemoimmunotherapy in selected relapsed patients but do not establish chemoimmunotherapy re-treatment as a preferred approach after modern frontline BR.

When chemoimmunotherapy is used after prior BR, current international guidelines generally favour a non-cross-resistant regimen, such as CHOP- or CVP-based therapy, rather than repeating bendamustine [5,12,13,14,15,16]. Retreatment with bendamustine is not recommended because of potential cumulative toxicities, including prolonged T-cell suppression, infections, and secondary malignancies [43]. Therefore, chemoimmunotherapy re-treatment should be individualized and generally reserved for patients with a lengthy prior remission, preserved marrow reserve, acceptable infection risk, and limited access to or unsuitability for novel-agent combinations.

**Recommendation 4.** Rituximab maintenance can be considered after repeat chemoimmunotherapy in patients who did not receive R-maintenance after first-line therapy.

**Consensus achieved:** 100% agreement (73% strongly agree, 27% agree); 91% of panelists supported inclusion of this recommendation.

**Strength of recommendation:** Grade 1B

**Rationale**

Given the absence of definitive randomized evidence, rituximab maintenance after repeat chemoimmunotherapy should be individualized. The panel considered this approach most appropriate for patients who did not previously receive rituximab maintenance after frontline therapy and who achieve a meaningful response to second-line chemoimmunotherapy as evidence supporting a second planned course of maintenance is limited and is not approved in any province. Potential benefits in disease control should be balanced against the risks of prolonged B-cell depletion, infections, and treatment burden [43].

**Recommendation 5.** The role of ASCT in relapsed/refractory FL without POD24 is uncertain in the era of targeted therapies and is not a preferred treatment approach.

**Consensus achieved:** 81% agreement (9% strongly agree, 45% agree, 9% neutral, 18% disagree, 18% agree once wording was revised); 80% of panelists supported inclusion of this recommendation.

**Strength of recommendation:** Grade 2C

**Rationale**

The role of ASCT in relapsed FL without POD24 is uncertain in the current era of targeted therapies. Randomized evidence is limited and largely derived from older treatment contexts (**See Clinical Question 4, recommendation 4** and Supplementary Tables S3 and S4). Retrospective analyses suggest the greatest benefit of ASCT may occur in patients with early treatment failure, while evidence in patients without POD24 is less compelling [44,45]. Given the lack of clear contemporary benefit, availability of effective novel-agent combinations, and potential for treatment-related morbidity and mortality, ASCT is not considered a preferred approach in late relapsed FL.

### 3.6. Therapeutic Approach in Third-Line and Later FL

**Clinical Question 6.** What is the optimal therapeutic approach for patients with R/R FL after ≥2 prior lines of systemic therapy?

**Recommendation 1.** CAR T-cell therapy is an effective option for patients with third-line and later FL, but has relatively high toxicity rates that make this a preferred option only for eligible patients.

**Consensus achieved:** 100% agreement (55% strongly agree, 45% agree); all panelists supported inclusion of this recommendation.

**Strength of recommendation:** Grade 2B

**Rationale**

CAR T-cell therapy has demonstrated substantial and durable efficacy in patients with heavily pretreated FL and represents an important treatment option in the third-line and later setting (Supplementary Tables S5 and S6). Three phase II studies evaluating tisagenlecleucel (ELARA) [46,47], axicabtagene ciloleucel (ZUMA-5) [48,49], and lisocabtagene maraleucel (TRANSCEND) [50] demonstrated high response rates and prolonged remissions in patients with R/R FL after multiple prior lines of therapy, including patients with POD24 and refractory disease.

In ELARA, tisagenlecleucel demonstrated an ORR of 86% and CR rate of 68%, with durable responses observed at long-term follow-up [46,47]. Updated analyses presented at ASTCT/CIBMTR 2026 demonstrated a median PFS of 53.2 months and a 5-year PFS rate of 46%, with similar outcomes observed in patients with POD24 (5-year PFS 41.1%) and double-refractory disease (5-year PFS 50.5%) [47]. OS outcomes were similarly favorable, with a 5-year OS rate of 74.1%. In ZUMA-5, axicabtagene ciloleucel achieved an ORR of 94% and CR rate of 79% in the FL population [48,49]. At 60 months, PFS was 49.8%, including 49.9% in patients with POD24 and 44.6% in refractory patients. Similarly, in TRANSCEND, lisocabtagene maraleucel demonstrated ORR and CR rates of 97% and 94%, respectively, with durable disease control maintained at longer follow-up, including in patients with POD24 and double-refractory disease [50]. Across the ELARA, ZUMA-5, and TRANSCEND FL studies, durable responses with CAR T-cell therapy were observed in high-risk subgroups, including patients with POD24 and refractory disease, suggesting that CAR T-cell therapy may mitigate some adverse prognostic features in relapsed/refractory FL.

Despite these favorable efficacy outcomes, CAR T-cell therapy is associated with clinically significant toxicity and substantial logistical complexity, including risks of CRS, neurologic events, prolonged cytopenias, infections, hypogammaglobulinemia, secondary malignancies, and the need for specialized centres with cellular therapy expertise. Rates and severity vary by product, with higher rates of CRS and neurologic toxicity reported with axicabtagene ciloleucel than with tisagenlecleucel or lisocabtagene maraleucel [46,47,48,49,50].

Given the excellent long-term disease control observed with CAR T-cell therapy, the panel considered CAR T-cell therapy an important treatment option for relatively fit patients with multiply relapsed FL. However, due to the risk of severe toxicity, treatment-related morbidity, and the need for specialized infrastructure and monitoring, the panel did not consider CAR T-cell therapy to be the preferred option for all patients in the third-line and later setting. Treatment selection should therefore be individualized based on patient fitness, comorbidities, disease progression, patient preference, caregiver support, and access to cellular therapy programs.

**Recommendation 2.** LenR triplets are effective options for patients, particularly those who have not been previously exposed to LenR.

**Consensus achieved:** 92% agreement (64% strongly agree, 0% agree, 9% neutral, 18% disagree, 9% other); 82% of panelists supported inclusion of this recommendation. All panelists agreed with the recommendation after a second survey where the recommendation was revised.

**Strength of recommendation:** Grade 2B

**Rationale**

As discussed above, tafasitamab-LenR and epcoritamab-LenR demonstrated improved PFS and response outcomes compared with LenR-based control arms (Supplementary Tables S5 and S6) [38,39]. Interpretation of these data should consider that patients with recent lenalidomide exposure were largely excluded from the pivotal triplet studies. Therefore, LenR triplets are considered most appropriate for patients who have not previously received LenR. However, there is no biological reason to think that patients who are not refractory to LenR should not be able to obtain benefit from LenR triplets. As there are no direct comparative studies between LenR triplets and BsAb monotherapy in the third-line and later setting, it remains uncertain whether the addition of LenR to BsAb therapy provides sufficient incremental benefit over BsAb monotherapy to justify the additional toxicity, cost, and treatment complexity in patients previously exposed to LenR. Treatment selection should therefore be individualised based on prior therapies, patient fitness, toxicity profile, logistical considerations, and access to therapy.

**Recommendation 3.** Bispecific antibody monotherapy is an effective option for third-line and later relapsed/refractory FL.

**Consensus achieved:** 90% agreement (45% strongly agree, 18% agree, 18% neutral, 9% strongly disagree, 9% other); 80% of panelists supported inclusion of this recommendation. 89% of panelists agreed with the recommendation after a second survey where the recommendation was revised.

**Strength of recommendation:** Grade 2B

**Rationale**

In the third-line setting, BsAbs, particularly mosunetuzumab, represent effective non-chemotherapy options with meaningful activity in heavily pretreated R/R FL (Supplementary Tables S5 and S6).

In the phase 1/2 study by Sehn et al., intravenous mosunetuzumab demonstrated durable activity in a heavily pretreated population with a median of 3 prior lines of therapy, including patients previously exposed to bendamustine (67%), ASCT (31%), PI3K inhibitors (19%), and immunomodulatory agents (14%) [9]. Approximately 52% of patients had POD24 and 69% were refractory to prior therapy. Mosunetuzumab achieved a median PFS of 24 months and 36-month PFS and OS rates of 43.2% and 82.4%, respectively. Similar efficacy was observed with subcutaneous mosunetuzumab in the phase 2 study by Bartlett et al., also in patients with a median of 3 prior lines of therapy [51]. Activity was maintained in high-risk subgroups, including patients with POD24 (ORR 73.2%, CR 56.1%, median PFS 18.5 months) and double-refractory disease (ORR 70.5%, CR 50%, median PFS 24 months). Mosunetuzumab was generally manageable from a safety perspective, with CRS occurring in 44.4% of patients, with grade 3/4 CRS in 2.2%, while no treatment-related grade 5 AEs were observed [9]. In the subcutaneous study, CRS occurred in 29.8% of patients (grade 3 in 2.1%) with no reported ICANS [51]. There are several BsAbs being tested in B-cell lymphomas with efficacy in R/R FL, but to date only mosunetuzumab has a monotherapy indication in Canada [52,53].

In addition to mosunetuzumab, other CD20 × CD3 BsAbs, including epcoritamab, glofitamab, and odronextamab, have demonstrated substantial clinical activity in R/R FL and other B cell malignancies, supporting the broader role of BsAbs in the evolving treatment landscape [53,54,55]. Although epcoritamab-LenR demonstrated excellent efficacy in EPCORE FL-1, including in patients with POD24 and double-refractory disease, this regimen includes lenalidomide and therefore may be less attractive in patients progressing after prior LenR-based therapy [39].

**Recommendation 4.** Zanubrutinib plus obinutuzumab can be considered for third-line and later relapsed/refractory FL patients.

**Consensus achieved:** 91% agreement (18% strongly agree, 73% agree, 9% disagree); 91% of panelists supported inclusion of this recommendation. 89% of panelists agreed with the recommendation after a second survey where the recommendation was revised.

**Strength of recommendation:** Grade 2B

**Rationale**

The phase 2 randomized ROSEWOOD study evaluated zanubrutinib plus obinutuzumab (ZO) versus obinutuzumab monotherapy in heavily pretreated (third-line and beyond) patients with R/R FL [56,57] (Supplementary Tables S5 and S6). ZO improved efficacy outcomes, including ORR (70.3% vs. 44.4%), CR rate (42.1% vs. 19.4%), and median PFS (22.1 vs. 10.3 months; HR 0.54, *p* = 0.0012), with activity also observed in high-risk subgroups such as POD24 and rituximab-refractory disease. Although OS numerically favored ZO, this difference was not statistically significant. The toxicity profile of ZO was generally manageable and consistent with BTK inhibitor therapy [56,57]. Grade 3/4 thrombocytopenia and neutropenia occurred in 15% and 24% of patients, respectively, while any-grade infections occurred in 55%. Rates of atrial fibrillation and hypertension were low (3% each), and treatment discontinuation due to adverse events occurred in approximately 10% of patients.

The combination improved PFS and received Health Canada approval; however, funding and access remain limited because obinutuzumab monotherapy is not considered a relevant comparator in Canada. Given the availability of other therapies, it remains unclear where ZO fits in the 3L+ treatment landscape in Canada.

### 3.7. CHCG and LLSC Perspective on Equity and Patient-Centred Care

Although these recommendations are organized primarily according to disease-related factors such as POD24 status, histologic transformation, and prior therapy, treatment selection in R/R FL should also be individualized according to patient-specific factors. This is particularly important as participants enrolled in clinical trials often represent a selected patient population and may not reflect the broader population encountered in routine clinical practice. Age, performance status, frailty, comorbidities, organ function, treatment goals, and patient preferences may substantially influence the balance between efficacy and tolerability.

Treatment-related toxicities should also be considered when selecting therapy and this should be weighed alongside expected efficacy when making individualized treatment decisions.

Treatment access and financial burden are also important considerations when selecting treatments for R/R FL as with other malignancies. Availability and reimbursement of novel therapies may vary across provinces and territories, and the logistical demands of treatment, including travel to specialized centres, time away from work, caregiver burden, and supportive care requirements, can affect treatment feasibility. As these recommendations were developed by Canadian experts for the Canadian healthcare system, differences in treatment availability, reimbursement, and financial toxicity across different health systems should be considered when applying these recommendations outside of Canada.

The CHCG and LLSC recognize that achieving consensus on evidence-based treatment recommendations is only one component of improving outcomes for patients with relapsed/refractory follicular lymphoma. Equally important is ensuring that advances in therapy can be accessed and implemented in a manner that is patient-centred and equitable across diverse Canadian healthcare settings.

Treatment decisions should incorporate not only disease characteristics and clinical evidence, but also patient preferences, treatment goals, geographic location, travel requirements, caregiver support, financial considerations, employment obligations, and access to specialized centres. The panel also recognizes that access to therapies may vary across Canada due to differences in provincial funding, reimbursement pathways, and local healthcare resources. While therapies such as CAR T-cell therapy, bispecific antibodies, and other novel treatments have expanded therapeutic options, differences in funding, availability, monitoring requirements, and treatment logistics may create barriers for some patients.

The CHCG and LLSC believe that national consensus recommendations can play an important role in supporting consistent, evidence-informed treatment and funding decisions across Canada. By providing a transparent assessment of the available evidence and expert consensus, these recommendations may help inform policy discussions and contribute to more equitable access to effective therapies regardless of geographic location.

Future efforts should continue to evaluate not only the efficacy and safety of therapies, but also their real-world feasibility, accessibility, and impact on health equity. Ensuring that therapeutic advances translate into meaningful benefits for all patients will require ongoing attention to equitable access, shared decision-making, and the lived experiences of patients navigating treatment across Canada.

## 4. Future Directions

Although current therapeutic options have markedly improved in recent years, there will still be patients who progress or relapse following available therapies. Clinical trial participation should be prioritized whenever feasible. In highly selected, fit patients with multiply relapsed disease and limited remaining treatment options, allogeneic HSCT may also be considered. Retrospective data from the EBMT demonstrated a 5-year OS of approximately 61% following allogeneic HSCT; however, this must be balanced against substantial toxicity, including a 5-year treatment-related mortality of approximately 19% [58].

The treatment landscape for R/R FL continues to evolve rapidly, and several promising therapies and combinations may further expand treatment options in Canada over the coming years. Increasing use of CD20-directed bispecific antibodies (BsAbs), including mosunetuzumab, epcoritamab, and potentially odronextamab, is expected to further shift treatment away from conventional chemotherapy in many patients. Additional funding and access to combination regimens such as epcoritamab-lenalidomide-rituximab (Epcor-LenR), tafasitamab-lenalidomide-rituximab (tafa-LenR), and zanubrutinib-obinutuzumab may substantially alter second-line and later-line treatment approaches. Ongoing studies are also evaluating earlier integration of CAR T-cell therapies including tisagenlecleucel, axicabtagene ciloleucel, and lisocabtagene maraleucel, as well as novel BsAb combinations and chemotherapy-free approaches. Improved understanding of treatment sequencing, biomarkers of response, mechanisms of resistance, and long-term toxicity will remain important areas of future research. As the therapeutic landscape continues to expand, future guideline updates will be required to incorporate emerging evidence and evolving Canadian access considerations.

## 5. Conclusions and Summary

The management of R/R FL has become increasingly complex with the rapid expansion of available therapies, including immunomodulatory combinations, BsAbs, CAR T-cell therapy, and other targeted approaches. As treatment options continue to evolve, clinicians must balance efficacy, toxicity, patient fitness, prior therapies, treatment goals, logistical considerations, and access to care within the Canadian healthcare system.

These CHCG recommendations were developed to provide practical, evidence-informed guidance for Canadian clinicians managing patients with R/R FL (Table 2). Key themes emerging from this consensus process include the importance of re-biopsy to exclude histologic transformation, individualized treatment selection based on timing of relapse and patient-specific factors, the growing role of novel immunotherapy-based combinations, and the evolving but increasingly selective role of transplantation strategies in the modern treatment era.

LenR-based triplet combinations incorporating epcoritamab or tafasitamab were identified as preferred treatment approaches in most patients with R/R disease in the second-line setting. In the third-line and later setting, CAR T-cell therapy and BsAbs (as monotherapy or in combination) represent important options.

These recommendations are limited by the absence of direct head-to-head comparisons among many contemporary treatment options, evolving regulatory and reimbursement status in Canada, and reliance on single-arm studies, subgroup analyses, or conference-updated data for several later-line therapies. Access to PET/CT, CAR T-cell therapy, BsAbs, and novel combination therapies also varies across provinces and institutions. Treatment decisions should therefore incorporate local availability and individual patient factors.

Given the pace of therapeutic advances in FL, these recommendations should be viewed as a contemporary framework that will continue to evolve as new clinical trial data, regulatory approvals, and real-world experience emerge. The CHCG hopes these recommendations will support clinicians in delivering evidence-based, patient-centered care for individuals with R/R FL across Canada.

## Figures and Tables

**Table 1:** Adapted British Committee for Standards in Haematology grading framework [11].

Strength of Recommendation	Quality of Evidence
**Strong (Grade 1):** Strong recommendations (Grade 1) are made when there is confidence that the benefits do or do not outweigh harm and burden. Grade 1 recommendations can be applied uniformly to most patients. Regard as “recommend”. **Weak (Grade 2):** Where the magnitude of benefit or not is less certain a weaker Grade 2 recommendation is made. Grade 2 recommendations require judicious application to individual patients. Regard as “suggest”.	**(A) High:** Further research is very unlikely to change confidence in the estimate of effect. Current evidence derived from randomised clinical trials without important limitations. **(B) Moderate:** Further research may well have an important impact on confidence in the estimate of effect and may change the estimate. Current evidence derived from randomised clinical trials with important limitations (e.g., inconsistent results, imprecision—wide confidence intervals or methodological flaws—e.g., lack of blinding, large losses to follow up, failure to adhere to intention to treat analysis), or very strong evidence from observational studies or case series (e.g., large or very large and consistent estimates of the magnitude of a treatment effect or demonstration of a dose–response gradient). **(C) Low:** Further research is likely to have an important impact on confidence in the estimate of effect and is likely to change the estimate. Current evidence from observational studies, case series or just opinion.

Adapted from Canadian Agency for Drugs and Technologies in Health. 2015. Available at: https://www.ncbi.nlm.nih.gov/books/NBK304620/ (accessed on 15 April 2026).

**Table 2:** Summary of CHCG recommendations for the treatment of R/R FL.

**INDICATIONS FOR TREATMENT**
**Clinical Question 1: When is therapy indicated for R/R FL?**
1. Asymptomatic patients with relapsed FL may be managed with active surveillance, with therapy deferred until symptomatic disease or progressive disease meeting GELF criteria.
2. Treatment for relapsed/refractory FL is recommended for any patient with symptomatic lymphoma.
**BASELINE EVALUATION PRIOR TO TREATMENT**
**Clinical Question 2. When should PET scans be considered?**
1. A PET scan should be considered in patients with concern for histologic transformation.
**ROLE OF RE-BIOPSY**
**Clinical Question 3. When should re-biopsy be performed?**
1. Re-biopsy should be considered if feasible in all patients with relapsed/refractory FL to confirm relapse and exclude histologic transformation.
**MANAGEMENT OF EARLY RELAPSE**
**Clinical Question 4. What is the optimal second-line therapy for FL patients who relapse within 2 years of chemoimmunotherapy +/− R-maintenance (POD24) or who are R-refractory?**
1. Patients who experience early recurrence of FL (POD24) should have a biopsy to exclude histologic transformation; patients with histologic transformation should be treated as per DLBCL recommendations.
2. LenR triplets (LenR + epcoritamab and LenR + tafasitamab) are recommended therapy for patients with early relapse of FL.
3. If chemoimmunotherapy is used for rituximab-refractory FL patients, obinutuzumab is the preferred monoclonal antibody.
4. Autologous stem cell transplantation can be considered for younger, fit patients with POD24 relapsed/refractory FL.
**MANAGEMENT OF LATE RELAPSE (>24 MONTHS)**
**Clinical Question 5: What is the optimal second-line therapy for FL patients who relapse more than 2 years following initiation of chemoimmunotherapy +/− R-maintenance?**
1. Patients who experience a late relapse of FL after prior chemoimmunotherapy have a number of possible therapy options. Decisions should be individualized based on patient factors.
2. Lenalidomide-rituximab (LenR) triplet combinations (LenR + epcoritamab or LenR + tafasitamab) are preferred over LenR.
3. Chemoimmunotherapy re-treatment (with an alternate chemotherapy) may be considered in patients who have experienced a very lengthy remission, but is not a preferred approach assuming the availability of newer combinations incorporating novel agents.
4. Rituximab maintenance can be considered after repeat chemoimmunotherapy in patients who did not receive R-maintenance after first-line therapy.
5. The role of ASCT in relapsed/refractory FL without POD24 is uncertain in the era of targeted therapies and is not a preferred treatment approach.
**THERAPEUTIC APPROACH IN THIRD-LINE AND LATER FL**
**Clinical Question 6: What is the optimal therapeutic approach for patients with R/R FL after ≥2 prior lines of systemic therapy?**
1. CAR T-cell therapy is an effective option for patients with third-line and later FL, but has relatively high toxicity rates that make this a preferred option only for eligible patients.
2. LenR triplets are effective options for patients, particularly those who have not been previously exposed to LenR.
3. Bispecific antibody monotherapy is an effective option for third-line and later relapsed/refractory FL.
4. Zanubrutinib plus obinutuzumab can be considered for third-line and later relapsed/refractory FL patients.

## Data Availability

No new datasets were generated or analyzed during the development of these consensus recommendations. Data sharing is therefore not applicable to this article.

## Supplementary Materials

The following supporting information can be downloaded at: https://www.mdpi.com/article/10.3390/curroncol33080451/s1, Table S1: Clinical Questions and Search Terms; Table S2: Studies evaluating SUVmax threshold in PET to identify histological transformation; Table S3: Baseline characteristics for key clinical trials evaluating second-line therapy for patients with FL; Table S4: Efficacy outcomes for key clinical trials evaluating second-line therapy for patients with FL; Table S5: Baseline characteristics for key clinical trials evaluating third-line therapy for patients with FL; Table S6: Efficacy outcomes for key clinical trials evaluating third-line therapy for patients with FL.
