# Supplementary material for: Canadian Hematology Consensus Group Recommendations for the Management of Relapsed and/or Refractory Follicular Lymphoma
Source: Curr Oncol. 2026 Jul 28;33(8):451. doi: 10.3390/curroncol33080451 (PMC13510464; doi:10.3390/curroncol33080451)
Supplement: Supplementary file 1 [file curroncol-33-00451-s001.zip › curroncol-4392867-supplementary.pdf]

Current Oncology

MDPI

Supplementary Materials

Table S1. Clinical Questions and Search Terms.

No.	Clinical Questions	Search Terms
1	When is therapy indicated for R/R FL?	
2	When should PET scans be considered?	MeSH terms: (lymphoma, follicular), (biopsy), (tomography, x-ray computed), (tomography, positron emission)<br><br>Text Words: (follicular lymphoma), (biopsy), (re-biopsy), (transformation), (CT), (computed tomography), (PET/CT), (Positron emission tomography)<br><br>Filters applied: past 10 years, human, english, adults<br><br>Publication type filters: Adaptive Clinical Trial, Clinical Study, Clinical Trial, Clinical Trial, Phase I, Clinical Trial, Phase II, Clinical Trial, Phase III, Clinical Trial, Phase IV, Comparative Study, Conference Proceedings, Consensus Statement, Controlled Clinical Trial, Editorial, Equivalence Trial, Guideline, Letter, Meta-Analysis, Multicenter Study, Network Meta-Analysis, Observational Study, Practice Guideline, Pragmatic Clinical Trial, Randomized Controlled Trial, Systematic Review, Validation Study.
3	When should re-biopsy be performed?	
4	What is the optimal second-line therapy for FL patients who relapse within 2 years after initiation of chemotherapy +/- R maintenance? (POD24)	Text Words: (relapsed follicular lymphoma), (treatment)<br><br>Filters applied: clinical trial, past 10 years, human, English, adults
5	What is the optimal second-line therapy for FL patients who relapse more than 2 years after initiation of chemotherapy +/- R maintenance?	
6	What is the best therapy option for a patient who has failed 2 or more lines of systemic therapy for FL?	

Table S2. Studies evaluating SUVmax threshold in PET to identify histological transformation.

Study, first author	Year	Design	Population/ Sample size	PET Threshold used	Sensitivity	Speci-ficity	Notes
**Sorigue M, et al [1]**	2026	System-atic review, meta-analysis	N/A	N/A	N/A	N/A	Area under the ROC curve for SUVmax to distinguish between FL/iNHL and HT ranged from 0.68 to 0.97. Sensitivity and specificity of the proposed cutoffs also varied widely.
**Lee ONY, et al [2]**	2025	System-atic review, meta-analysis	N/A	N/A	N/A	N/A	See included studies below; Authors' discussion: High study variability and insufficient evidence

							prevented a meta-analysis of findings for either the FL or mixed histological population groups.
**Bodet-Milin C, et al [3] (In Lee and Sorigue review)**	2008	Prospective cohort	FL = 23	SUVmax 14	0.90 (0.55-1.00)	0.85 (0.55-0.98)	
**Rajamaki A, et al [4] (In Lee and Sorigue review)**	2023	Retrospective cohort	FL = 63	SUVmax 26.5	0.86 (0.42-1.00)	1.00 (0.94-1.00)	Note: Only patients with SUVmax ≥ 10 were biopsied, thus individuals with SUVmax < 10 were excluded when calculating diagnostic performance
**Wondergem MJ, et al [5] (In Lee review)**	2015	Prospctive, case-control	FL = 26	SUVmax 14.5	1.00 (0.66-1.00)	0.82 (0.57-0.96)	Note: Also looked at high TLG to detect transformation in a mixed histological subtype population.
**Obeid JP, et al [6] (In Lee review)**	2023	Retrospective case-control	FL = 83; Non-FL = 23	SUVmax 10	0.88 (0.76-0.95)	0.74 (0.60-0.85)	Limitation: population not exclusively FL
**Wai SH, et al [7] (In Lee and Sorigue review)**	2024	Retrospective cohort	FL = 54; CLL/SLL = 14; MZL = 14; Other = 4	SUVmax 12 or 15 or 25	0.71 (0.52-0.86) or 0.45 (0.27-0.64) or 0.26 (0.12-0.45)	0.58 (0.44-0.72) or 0.74 (0.60-0.85) or 0.96 (0.87-1.00)	Limitation: population not exclusively FL
**Schoder H, et al [8] (In Sorigue review)**	2005	Retrospective cohort	NHL = 97	SUVmax >10	0.63	NR	Limitation: population not exclusively FL
**Noy A, et al [9] (In Sorigue review)**	2009	Retrospective cohort	FL = 22	SUVmax >10<br>SUVmax >13 0.57	0.71<br>0.57	NR	Limitation: population not exclusively FL
**Shichijo T, et al [10] (In Sorigue review)**	2020	Retrospective cohort	R/R FL = 76	SUVmax ≥10<br>SUVmax ≥16<br>SUVmax ≥20	0.86–1<br>0.59–0.67<br>0.22–0.67	0.37–0.57<br>0.79–0.96<br>0.95–1	Limitation: population not exclusively FL

Table S3. Baseline characteristics for key clinical trials evaluating second-line therapy for patients with FL.

Study	Design	Treatment	Sample size: Total (T)	Sample size: FL population	Prior therapy: Median (range)	Prior therapy: % 1 prior Tx	Prior therapy: % prior BR	% POD24	Refractory: Last line	Refractory: Rituximab	Refractory: Double	Median Age (range)	High tumour burden (GELF)	FLIPI 3-5	LDH >ULN
Lenalidomide + anti-CD20 monoclonal antibodies															
AUGMENT Leonard 2019 [11]	Ph 3, R, Double-blind	R2 vs Pb-R	358	295	1 (1-12)	56%	9% (Chemo-R: 72%)	R2: 31%[1] Pb-R: 34%[1]	R2: 17% Pb-R: 14%	Not reported	Not reported	63 (26-88) ≥65: 43%	51% Bulky disease: 26%	34%	23%
Anti-CD20 bispecific T cell engagers															
EPCORE FL-1 Falchi 2026 [12]	Ph 2, R, open-label	Epcor-R2 vs R2	488	488	1 (IQR 1-2)	Epcor-R2: 60% R2: 58%	Not reported[2]	Epcor-R2[3]: 44% R2[3]: 38%	Epcor-R2: 35% R2: 33%	Epcor-R2: 43% R2: 42%	Epcor-R2: 37% R2: 37%	Epcor-R2: 60 (50-69) ≥65: 36% R2: 63 (54-71) ≥65: 43%	NR Bulky disease Epcor-R2: 19% R2: 25%	Epcor-R2: 41% R2: 46%	Not reported
Anti-CD19 therapies															
inMIND Sehn 2026 [13]	Ph 3, R, Double-blind	Tafa-R2 vs Pb-R2	548	548	1 (1-7)	55%	Not reported (B or BR: 39%)	Tafa-R2[1]: 31% Pb-R2[1]: 32%	Tafa-R2: 41% Pb-R2: 35%	Tafa-R2: 43% Pb-R2: 42%	Not reported	64 (31-88) ≥75: 20%	83%	52%	Not reported
Chemoimmunotherapy															
GADOLIN Cheson 2018 [14]	Ph 3, R, open-label	BO vs B	413	335	1	Not reported	N/A	Not reported	FL population: BO: 94% B:92%	100%	FL population: BO: 79% B:80%	63 (34-87)	Bulky disease: 34%	40%	Not reported
StiL NHL 2-2003 Rummel 2016 [15]	Ph 3, R, open-label	BR vs FR (Protocol amendment to allow R maintenance	219	111 (BR 58; FR 53)	Total pop: 1 (IQR 1-2)	Total pop: BR: 66% FR: 51%	Total pop: N/A[5]	NR	Total pop: 5%	N/A	N/A[4]	Total pop: BR: 68.5 (IQR 59-74) ≥70: 38%	Total pop: NR Bulky disease: BR: 20%	Total pop: BR: 43% FR: 40%	Total pop: BR: 34% FR: 40%

		in responders)										FR: 66.4 (IQR 59-74) ≥70: 35%%	FR: 24%		
Autologous stem cell transplant															
FLAZ12 Ladetto 2024 [16] [6]	Ph 3, R, open-label	ASCT vs RIT	141 (Randomized)	141 (Randomized)	Not reported	90%	Not reported (Benda or F: 15%; Chemo-R: 98%)	33%[1]	8%	N/A	N/A	57 (48-62)	Not reported Bulky disease: 81%	Not reported	23%
Schouten 2003 [17]	Ph ?, R, open-label	Chemo [C] vs. HDT+ASCT (purged [P] and unpurged [U])	70	70	Not reported	69%	N/A	Not reported	Not reported	N/A	N/A	47 (30-64)	Not reported	Not reported	Not reported

[1]Definition of POD24 = progression of disease within 24 months of diagnosis.

[2]The sample of participants with recent bendamustine use within 12 months was too small (seven participants) to provide meaningful conclusions on benefit of Epcor-R2 in this subgroup.

[3]Definition of POD24 = progression of disease within 24 months of first-line chemoimmunotherapy.

[4]Patients refractory to regimens that included rituximab, bendamustine, or purine analogue drugs were excluded.

[5]Prior bendamustine in 11% in the BR arm and 13% in the FR arm. Prior rituximab in 39% in the BR arm and 45% in the FR arm.

[6]Study ended early due to low enrollment.

Abbreviations: ASCT = autologous stem cell transplantation; B = bendamustine; BO = Bendamustine-obinutuzumab; BR = bendamustine-rituximab; Chemo-R = chemotherapy + rituximab; Epcor = epcoritamab; F = fludarabine; FL = follicular lymphoma; FLIPI = Follicular Lymphoma International Prognostic Index; FR = fludarabine-rituximab; GELF = Groupe d'Etude des Lymphomes Folliculaires; HDT = high-dose therapy; IQR, interquartile range; LDH = lactate dehydrogenase; N/A = not applicable; NR = not reached; P = purged; Pb = placebo; Ph = phase; POD24 = progression of disease within 24 months; R = randomized; R2 = rituximab + lenalidomide; RIT = radioimmunotherapy;Tafa = tafasitamab; Tx = treatment; U = unpurged; ULN = upper limit of normal.

Table S4. Efficacy outcomes for key clinical trials evaluating second-line therapy for patients with FL.

Study	Treatment	Median follow-up	Primary end-point	Response						PFS/OS					
				ORR		CR		Odds ratio		Median (months)		Landmark		HR (95% CI); p-value	
				Overall	Subgroup	Overall	Subgroup	Overall	Subgroup	Overall	Subgroup	Overall	Subgroup	Overall	Subgroup
Lenalidomide + anti-CD20 monoclonal antibodies															
AUGMENT [11]	R2 vs Pb-R	28.3 months	PFS (R2 vs Pb) in total population: HR 0.46; 95% CI 0.34-0.62. Median 39.4 vs 14.1 months.	FL group:<br><br>R2: 80%<br>Pb-R: 82%<br><br>P<0.0001	Not reported	FL group:<br><br>R2: 51%<br>Pb-R: 29%<br><br>P=0.004	Not reported	Not reported	Not reported	FL group:<br><br>R2: 39.4<br>Pb-R: 13.9<br><br>Prior BR subgroup (R2, n=19 vs Pb-R, n=14):<br><br>R2: NR<br>Pb-R: 11.1	Not reported	PFS<br>2- year (Total population):<br>R2: 58%<br>Pb-R: 36%<br><br>OS<br>2-year (Total population):<br>R2: 93%<br>Pb-R: 87%	Not reported	FL group (R2 vs Pb-R):<br><br>PFS<br>0.40 (0.29-0.56); p<0.0001<br><br>OS<br>0.45 (0.22-0.92); P = 0.02	PFS<br>Prior BR subgroup (R2, n=19 vs Pb-R, n=14):<br><br>0.23 (0.06-0.85)
Anti-CD20 bispecific T cell engagers															

EPCORE FL-1 [12]	Epcor-R2 vs R2	14.8 months	Dual primary endpoint (superiority): PFS and ORR	Epcor-R2: 95%<br><br>R2: 75%	Not reported	Epcor-R2: 83%<br><br>R2: 50%	POD24 Epcor-R2: 80.3%<br><br>R2: 38.7%<br><br>Double-refractory Epcor-R2: 79.1%<br><br>R2: 35.2%	Not reported	Not reported	PFS Epcor-R2: NR<br><br>R2: 11.7 months<br><br>OS NR both arms	PFS POD24: Epcor-R2: NR<br><br>R2: 9.6 months<br><br>Double-refractory: Epcor-R2: 21.9 months<br><br>R2: 10.9 months	PFS Not reported<br><br>OS 16-month: Epcor-R2 95.8%<br><br>R2: 88.8%	Not reported	PFS (Epcor-R2 vs R2):<br><br>0.21 (0.14-0.31)	PFS POD24: (Epcor-R2 vs R2):<br><br>0.22 (0.13-0.37)<br><br>Double-refractory: (Epcor-R2 vs R2):<br><br>0.23 (0.13-0.39)
Anti-CD19 therapies															
inMIND [13]	Tafa-R2 vs Pb-R2	14.1 months	PFS (Tafa-Pb-R2 vs Pb):<br><br>HR 0.43; 95% CI 0.32-0.58<br><br>Median 22.4 vs 13.9 months	Tafa-R2: 84%<br><br>Pb-R2: 72%	Not reported	Tafa-R2: 45%<br><br>Pb-R2: 34%	Not reported	2.0 (1.30–3.02); p=0.0014	Not reported	PFS Tafa-R2: 22.4 Pb-R2: 13.9<br><br>OS Tafa-R2: NR Pb-R2: NR	PFS POD24: Tafa-R2: 19.2 Pb-R2: 11.3	Not reported	Not reported	(Tafa-R2 vs Pb-R2):<br><br>PFS 0.43 (0.32-0.58)<br><br>OS 0.59 (0.31-1.13)	(Tafa-R2 vs Pb-R2):<br><br>PFS POD24: 0.43 (0.27-0.69)<br><br>Refractory to anti-CD20: 0.44 (0.30-0.65)
Chemoimmunotherapy															
GADOLIN [14]	BO vs B	31.8 months	PFS Total pop. (BO vs B)	FL pop.	Not reported	Not reported	Not reported	Not reported	Not reported	FL pop.: PFS	Not reported	FL pop. PFS	FL pop. PFS	FL pop.	FL pop. (BO vs B)

			HR 0.57; 95% CI, 0.44 to 0.73; P<0.001<br>Median 25.8 months vs 14.1 months	*End of induction<br>BO:67.7%<br>O: 65.3%						BO: 25.3<br>B 14<br><br>OS<br>BO: NR<br>B: 53.9		1-yr:<br>BO: 69%<br>B: 55%	Double-refractory<br>1-yr:<br>BO: 67%<br>B: 51%<br>R mono-refractory<br>1-yr:<br>BO: 78%<br>B: 68%	(BO vs B)<br><br>PFS<br>0.52<br>(0.39-0.69); p <0.001<br><br>OS<br>0.58<br>(0.39-0.86); p = 0.006	PFS<br>Double-refractory<br>0.56 (0.41-0.75)<br><br>R mono-refractory<br>0.37 (0.17-0.79)
StiL NHL 2-2003 [15]	BR vs FR (Protocol amendment to allow R maintenance in responders)	96 months	PFS in total NHL population: (BR vs FR)<br><br>HR 0.56, (95% CI 0.34–0.87); p<0.0001<br><br>Median<br>BR: 34.2 months<br>FR: 11.7 months	Total pop.<br><br>BR: 82%<br>FR: 51%<br><br>P<0.0001	Total pop.<br><br>Patients previously treated with Benda and randomized to BR (n=13): 77%	Total pop.<br><br>BR:40%<br>FR:17%<br><br>P=0.0002	Total pop.<br><br>Patients previously treated with Benda and randomized to BR (n=13): 15%	Not reported	Not reported	PFS<br>FL pop:<br><br>BR: 54.5<br>FR: 22.9<br><br>OS<br>Total pop:<br>BR: 109.7<br>FR: 49.1	N/A	PFS<br>Total pop.<br><br>1-yr:<br>BR: 76%<br>FR: 48%	N/A	PFS<br>FL pop:<br>(BR vs FR)<br><br>0.56<br>(0.34–0.87)<br><br>OS<br>Total pop:<br>(BR vs FR)<br><br>0.64<br>(0.45-0.91);<br>p=0·012	N/A

Autologous stem cell transplant															
FLAZ12 [16]	ASCT vs RIT	77 months	See PFS	Not reported	Not reported	Not reported	Not reported	Not reported	Not reported	PFS not reported<br><br>OS ASCT: NR<br>RIT: NR	Not reported	PFS 3-yr:<br><br>ASCT: 62%<br>RIT: 62%<br><br>PFS 6-yr:<br>ASCT: 46%<br>RIT: 52%<br><br>OS 3-yr:<br><br>ASCT: 87%<br>RIT: 86%<br><br>OS 6-yr:<br>ASCT: 76%<br>RIT: 78%	Not reported	(ASCT vs RIT)<br><br>PFS: 1.11 (0.69-1.80); p=0.6662<br><br>OS: 0.94 (0.45-1.94); P = 0.8588	PFS POD24 (ASCT vs RIT):<br><br>0.84 (0.41-1.73)
Schouten [17]	Chemo [C] vs. HDT+ASCT (purged [P] and unpurged [U])	69 months	See PFS	Not reported	Not reported	Not reported	Not reported	Not reported	Not reported	Not reported	Not reported	PFS Not reported<br><br>OS 5-yr: 50% overall (not by treatment arm)	Not reported	(U/P [46 pts] vs. C [24 pts])<br><br>PFS: 0.30 (0.15-0.61); p =0.0009<br><br>OS: 0.40 (0.18-0.89); p =0.026	Not reported

Abbreviations: ASCT = autologous stem cell transplantation; B = bendamustine; BO = Bendamustine-obinutuzumab; BR = bendamustine rituximab; Chemo-R = chemotherapy + rituximab; CI = confidence interval; CR = complete response; Epcor = epcoritamab; F = fludarabine; FL = follicular lymphoma; HDT = high-dose therapy; HR = hazard ratio; IQR, interquartile range; mono = monotherapy; N/A = not applicable; NHL = non-Hodgkin lymphoma; NR = not reached; ORR = overall response rate; OS = overall survival; P = purged; Pb = placebo; PFS = progression-free survival; Ph = phase; POD24 = progression of disease within 24 months; R = rituximab; R2 = rituximab + lenalidomide; RIT = radioimmunotherapy; Tafa = tafasitamab; Tx = treatment; U = unpurged.

Table S5. Baseline characteristics for key clinical trials evaluating third-line therapy for patients with FL.

Study	Design	Treatment	Sample size		Prior therapy			% POD24	Refractory			Median Age (range)	High tumour burden (GELF)	FLIPI 3-5	LDH >ULN
			Total (T)	FL population	Median (range)	% 2 prior Tx	% prior Tx types		Last line	Rituximab	Double				
CAR T cell therapy															
ELARA Dreyling 2024 [18]	Ph 2, single-arm	Tisagenlecleucel	97	97	4 (2-13)	100% >4 lines: 28%	ASCT: 36%	63%[3]	78%	87%	68%	57 (IQR 49–64) ≥65: 25%	Not reported Bulky disease: 64%	60%	Not reported
ZUMA-5 Neelapu 2025 [19]	Ph 2, single-arm	Axicabtagene ciloleucel	159	127	3 (1-10)	Not reported ≥3 prior: 64%	FL pop.: ASCT 24% Single-agent anti-CD20 31% PI3Ki 27% Len 31%	Total pop,: 57%[3] FL pop.: 56%[3]	Total pop: 71% FL pop.: 69%	Not reported	Not reported	60 (34-79) ≥65: 34%	Not reported Bulky disease 52%	44%	Not reported
TRANSCEND Morschhauser 2024 [20]	Ph 2, single-arm	Lisocabtagene maraleucel	130	130	2 (1-10)	82% 2L (n=23) 3L+ (n=107)	Prior ASCT 25% (3L+, 31%; 2L, 0%), Prior B 55% (3L+, 61%; 2L, 26%)	56% (54% 3L+, 65% 2L)[3]	Not reported	Not reported	62% (3L+ 64%; 2L 48%)	3L+: 62 (23–80) 2L: 53 (34–69)	56% (3L+ 53%; 2L 70%)	53% (3L+ 57%; 2L 35%)	41% (3L+ 44%; 2L 26%)
Anti-CD20 bispecific T cell engagers															
EPCORE FL-1 Falchi 2026 [12]	Ph 2, R, open-label	Epcor-R2 vs R2	488	488	1 (IQR 1-2)	25%	Anti-CD20 100%; CIT 98%; IMID 4%	Epcor-R2[3]: 44% R2[3]: 38%	Epcor-R2: 35% R2: 33%	Epcor-R2: 43% R2: 42%	Epcor-R2: 37% R2: 37%	Epcor-R2: 60 (50-69) ≥65: 36%	Not reported Bulky disease	Epcor-R2: 41% R2: 46%	Not reported

												R2: 63 (54-71) ≥65: 43%	Epcor-R2: 19% R2: 25%		
ELM-2 Kim 2024 [21]	Ph 2, single arm	odronextamab	128	128	3 (2-13)	NR ≥3 54%	PI3Ki 14%; R2 13%; ASCT 30%	49%[2]	69%	79% (previous anti-CD20)	41%	61.0 (22-84)	Not reported Bulky disease 14%	58%	NR
Sehn et al 2025 [22]	Ph 1/2, single arm	mosunetuzumab	90	90	3 (2-10)	39%	ASCT 31%; CART 3.3%; anthracyclines 82%; PI3Ki 19%; BTKi 7%; IMID 14% B 67%	52.2%[2]	69%	79% (anti-CD20)	53%	60 (29-90) >65 31%	Not reported Bulky disease 34%	44%	NR
Bartlett et al 2026 [23]	Ph 2, single arm	mosunetuzumab SC	94	94	3 (2-8)	46%	BTKi 6.4%; CAR T 4.3%; IMID 26.6% PI3Ki 11.7%	43.6%[2]	62.8%	66% (anti-CD20)	46.8%	65 (35–84)	Not reported Bulky disease 23%	55%	NR
Anti-CD19 therapies															
inMIND Sehn 2026 [13]	Ph 3, R, Double-blind	Tafa-R2 vs Pb-R2	548	548	1 (1-7)	25%	anthracyclines 50%; Anti-CD20+ CHOP 57%; B/BR/BO 39%; anti-CD20 mono 19%; anti-CD20 +CVP 13%; Chemo + ASCT 8%	Tafa-R2[1]: 31% Pb-R2[1]: 32%	Tafa-R2: 41% Pb-R2: 35%	Tafa-R2: 43% Pb-R2: 42%	Not reported	64 (31-88) ≥75: 20%	83%	52%	Not reported

BTK inhibitors															
ROSEWOOD Zinzani 2023 [24]	Ph 2, R (2:1), open-label	ZO vs O	217	217	3 (2-11)	NR 2 or 3 lines 73%	CIT 99%; Rituximab 100%; Obinutuzumab 6%; Anthracyclines 81%; bendamustine 55%; PI3Ki 20%; Lenalidomide 10%; autoSCT19%; alloSCT 2%	ZO 34%[2] O 42%[2]	ZO 32% O 40%	ZO 54% O 50%	NR	64.0 (31-88)	57%	53%	36%

[1]Definition of POD24 = progression of disease within 24 months of diagnosis.

[2]Definition of POD24 = progression of disease within 24 months of any first-line therapy.

[3]Definition of POD24 = progression of disease within 24 months of first-line chemoimmunotherapy.

Abbreviations: 2L = second-line; 3L+ = third or more line; ASCT = autologous stem cell transplantation; B = bendamustine; BR = bendamustine rituximab; BTKi, Bruton's tyrosine kinase inhibitor; CAR T = chimeric antigen receptor T cell therapy; Chemo-R = chemotherapy + rituximab; CIT = chemoimmunotherapy; Epcor = epcoritamab; F = fludarabine; FL = follicular lymphoma; FLIPI = Follicular Lymphoma International Prognostic Index; FR = fludarabine-rituximab; GELF = Groupe d'Etude des Lymphomes Folliculaires; HDT = high-dose therapy; IMID = immunomodulatory imide drugs; IQR, interquartile range; LDH = lactate dehydrogenase; Len = lenalidomide; N/A = not applicable; NR = not reached; P = purged; Pb = placebo; Ph = phase; POD24 = progression of disease within 24 months; PI3Ki = phosphatidylinositol 3-kinase inhibitor; R = randomized; R2 = rituximab + lenalidomide; RIT = radioimmunotherapy;Tafa = tafasitamab; Tx = treatment; U = unpurged; ULN = upper limit of normal.

Table S6. Efficacy outcomes for key clinical trials evaluating third-line therapy for patients with FL.

Study	Treatment	Median follow-up	Primary endpoint	Response			PFS/OS		
				ORR	CR	Odds ratio	Median (months)	Landmark	HR (95% CI); p-value

				**Overall**	**Subgroup**	**Overall**	**Subgroup**	**Overall**	**Subgroup**	**Overall**	**Subgroup**	**Overall**	**Subgroup**	**Overall**	**Subgroup**
CAR T cell therapy															
ELARA [18]	Tisagenlecleucel	29 months<br><br>ASTCT/ CIBMTR 2026 update: >5 years	See CR	86.2%	Refractory to last line: 85%<br><br>Double-refractory: 85%<br><br>POD24: 82%	68.1%	Refractory to last line: 69%<br><br>Double-refractory: 59%<br><br>POD24: 66%	N/A	N/A	PFS: NR<br>OS: NR<br><br>ASTCT/ CIBMTR 2026 update<br>PFS: 53.2 months<br>OS: NR	Not reported	PFS 2yr: 57.4%<br>OS 2yr: 87.7%<br><br>ASTCT/ CIBMTR 2026 update:<br>PFS 5-yr: 46%<br>OS 5-yr: 74.1%	ASTCT/ CIBMTR 2026 update:<br><br>POD24:<br>PFS 5-yr: 41.1%<br>OS 5-yr: 74.6%<br><br>Double refractory:<br>PFS 5-yr: 50.5%<br>OS 5-yr: 79.8%	N/A	N/A
ZUMA-5 [19]	Axicabtagene ciloleucel	64.4 months	ORR in iNHL pop: 90% (CR 75%)	FL pop: 94%	Not reported	FL pop: 79%	Not reported	N/A	N/A	FL pop: PFS: 57.3<br>OS: Not reported	Not reported	FL pop: PFS 60-month: 49.8%<br>OS 60-month: 69%	FL pop PFS 60-month:<br><br>Refractory: 44.6%<br><br>POD24: 49.9%	N/A	N/A
TRANSCEND [20]	Lisocabtagene maraleucel	18.9 months	See ORR	97% (3L+ 97%; 2L 96%)	For 3L+:<br><br>Double-refractory: 96%	94% (3L+ 94%; 2L 96%)	For 3L+:<br><br>Double-refractory: 94%	N/A	N/A	PFS: NR<br>OS: NR	Not reported	PFS 12-month: 81% (3L+ 81%; 2L 91%)	For 3L+ 12-month PFS:	N/A	N/A

		ASH 2025 update on subgroup of 3L+<br><br>41.5 months		ASH 2025 update on subgroup of 3L+	POD24: 96%		POD24: 95%					OS 12-month: 93% (3L+ 92%; 2L 96%)<br><br>ASH 2025 update on subgroup of 3L+<br>PFS 36-month: 68%<br>OS 36-month: 86%	Double-refractory: 75%<br><br>POD24: 76%		
**Anti-CD20 bispecific T cell engagers**															
EPCORE FL-1 [12]	Epcor-R2 vs R2	14.8 months	Dual primary endpoint (superiority): PFS and ORR	Epcor-R2: 95%<br><br>R2: 75%	Not reported	Epcor-R2: 83%<br><br>R2: 50%	POD24 Epcor-R2: 80.3%<br><br>R2: 38.7%<br><br>Double-refractory Epcor-R2: 79.1%<br><br>R2: 35.2%	Not reported	Not reported	PFS Epcor-R2: NR<br><br>R2: 11.7 months<br><br>OS NR both arms	PFS POD24: Epcor-R2: NR<br><br>R2: 9.6 months<br><br>Double-refractory: Epcor-R2: 21.9 months	PFS Not reported<br><br>OS 16-month: Epcor-R2 95.8%<br><br>R2: 88.8%	Not reported	PFS (Epcor-R2 vs R2):<br><br>0.21 (0.14-0.31)	PFS POD24: (Epcor-R2 vs R2):<br><br>0.22 (0.13-0.37)<br><br>Double-refractory: (Epcor-R2 vs R2):

											R2: 10.9 months				0.23 (0.13-0.39)
ELM-2 [21]	odronextamab	20.1 months	See ORR	80%	POD24: 81%<br><br>Refractory to last line: 78.3%<br><br>Anti-CD20 refractory: 78.9%<br><br>Double-refractory: 73.6%	73.4%	POD24: 73%	N/A	N/A	PFS 20.7<br><br>OS NR	Not reported	PFS 12-month: 66%<br><br>24-month: 46%<br><br>OS 12-month: 86%<br><br>24-month 70%	Not reported	N/A	N/A
Sehn et al 2025 [22]	mosunetuzumab	37.4 months	See CR	77.8%	Not reported[1]	60%	Not reported[1]	N/A	N/A	PFS 24<br><br>OS NR	Not reported	PFS 36-month: 43.2%<br><br>OS 36-month: 82.4%	Not reported	N/A	N/A
Bartlett et al 2026 [23]	mosunetuzumab SC	26.1 months	PK endpoints (non-inferiority C_{trough} and	76.6%	POD24 73.2%<br><br>Double-refractory: 70.5%	61.7%	POD24 56.1.2%<br><br>Double-refractory: 50%	N/A	N/A	PFS 23.7<br><br>OS NR	PFS POD24: 18.5	PFS 18-month: 56.8%	PFS POD24: 53.8%	N/A	N/A

			AUC vs IV admin)								Double-refractory: 24		Double-refractory: 50.1%		
Anti-CD19 therapies															
inMIND [13]	Tafa-R2 vs Pb-R2	14.1 months	PFS (Tafa-Pb-R2 vs Pb): HR 0.43; 95% CI 0.32-0.58 Median 22.4 vs 13.9 months	Tafa-R2: 84% Pb-R2: 72%	Not reported	Tafa-R2: 45% Pb-R2: 34%	Not reported	2.0 (1.30–3.02); p=0.0014	Not reported	PFS Tafa-R2: 22.4 Pb-R2: 13.9 OS Tafa-R2: NR Pb-R2: NR	PFS POD24: Tafa-R2: 19.2 Pb-R2: 11.3	Not reported	Not reported	(Tafa-R2 vs Pb-R2): PFS 0.43 (0.32-0.58) OS 0.59 (0.31-1.13)	(Tafa-R2 vs Pb-R2): PFS POD24: 0.43 (0.27-0.69) Refractory to anti-CD20: 0.44 (0.30-0.65)
BTK inhibitors															
ROSEWOOD [24] +UPDATE from ASH 2025 [25]	ZO vs O	20.2 months Update from ASH 2025: 34.6 months	See ORR	ZO 69% O 46% P=0.001 Update from ASH 2025: ZO 70.3% O 44.4% P=0.003	POD24: ZO 60% O:47% Rituximab-refractory: ZO 62% O 38% Update from ASH 2025: POD24: ZO 63% O:43%	ZO 39% O 19% P=0.004 Update from ASH 2025: ZO 42.1% O 19.4% P=0.009	Not reported	Not reported	Not reported	PFS: ZO: 28 O: 10.4 OS: ZO: NR O: 34.6 Update from ASH 2025: PFS: ZO: 22.1 O: 10.3 months	Not reported	PFS NR OS 24-month: ZO: 77% O:71%	Not reported	(ZO vs O) PFS 0.50 (0.33 to 0.75); p<0.001 OS 0.62 (0.35 to 1.07); p=0.085	Not reported

	Rituximab-refractory:<br>ZO 62%<br>O 36%	OS:<br>ZO: NR<br>O: 41.2	Update from ASH 2025:<br>PFS: 0.54 (0.37-0.79); p=0.0012<br><br>OS: 0.66 (0.43-1.04); p=0.0698

[1]Note subgroup analysis of larger study found ORR/CR to be numerically higher in older vs younger patients Thiruvengadam 2026.

Abbreviations: ASCT = autologous stem cell transplantation; AUC = area under curve; B = bendamustine; BO = Bendamustine-obinutuzumab; BR = bendamustine rituximab; Chemo-R = chemotherapy + rituximab; CI = confidence interval; CR = complete response; Epcor = epcoritamab; F = fludarabine; FL = follicular lymphoma; HDT = high-dose therapy; HR = hazard ratio; IQR, interquartile range; mono = monotherapy; IV = intravenous; N/A = not applicable; NHL = non-Hodgkin lymphoma; NR = not reached; O = obinutuzumab; ORR = overall response rate; OS = overall survival; P = purged; Pb = placebo; PFS = progression-free survival; Ph = phase; POD24 = progression of disease within 24 months; R = rituximab; R2 = rituximab + lenalidomide; RIT = radioimmunotherapy; Tafa = tafasitamab; Tx = treatment; U = unpurged; ZO = zanubrutinib-obinutuzumab.

References

1. Sorigue, M.; Miljkovic, M.; Mozas, P. Letter to the Editor regarding 18F-FDG PET or PET/CT in detecting high-grade transformation of chronic lymphocytic leukaemia and indolent lymphomas: A systematic review and meta-analysis. *Br. J. Radiol.* **2025**, *99*, 583–584. https://doi.org/10.1093/bjr/tqaf144.
2. Lee, O.N.Y.; Kuruvilla, J.; Hodgson, D.C.; Veit-Haibach, P.; Metser, U. 18F-FDG PET or PET/CT in detecting high-grade transformation of chronic lymphocytic leukaemia and indolent lymphomas: A systematic review and meta-analysis. *Br. J. Radiol.* **2025**, *98*, 669–678. https://doi.org/10.1093/bjr/tqaf028.
3. Bodet-Milin, C.; Kraeber-Bodéré, F.; Moreau, P.; Campion, L.; Dupas, B.; Le Gouill, S. Investigation of FDG-PET/CT imaging to guide biopsies in the detection of histological transformation of indolent lymphoma. *Haematologica* **2008**, *93*, 471–472. https://doi.org/10.3324/haematol.12013.
4. Rajamäki, A.; Kuitunen, H.; Sorigue, M.; Kokkonen, S.M.; Kuittinen, O.; Sunela, K. FDG-PET/CT-guided rebiopsy may find clinically unsuspicious transformation of follicular lymphoma. Cancer Med. 2023;12:407-11.
5. Wondergem, M.J.; Rizvi, S.N.; Jauw, Y.; Hoekstra, O.S.; Hoetjes, N.; van de Ven, P.M.; Boellaard, R.; Chamuleau, M.E.; Cillessen, S.A.; Regelink, J.C.; et al. ^{18}F-FDG or 3′-Deoxy-3′-^{18}F-Fluorothymidine to Detect Transformation of Follicular Lymphoma. *J. Nucl. Med.* **2015**, *56*, 216–221. https://doi.org/10.2967/jnumed.114.149625.
6. Obeid, J.; Hiniker, S.M.; Schroers-Martin, J.; Guo, H.H.; No, H.J.; Moding, E.J.; Advani, R.H.; Alizadeh, A.A.; Hoppe, R.T.; Binkley, M.S. Investigating and modeling positron emission tomography factors associated with large cell transformation from low-grade lymphomas. *eJHaem* **2022**, *4*, 90–99. https://doi.org/10.1002/jha2.615.
7. Wai, S.H.; Lee, S.T.; Cliff, E.R.S.; Bei, M.; Lee, J.; Hawkes, E.A.; Chong, G. Utility of FDG-PET in predicting the histology of relapsed or refractory lymphoma. *Blood Adv.* **2024**, *8*, 736–745. https://doi.org/10.1182/bloodadvances.2023011566.
8. Schöder, H.; Noy, A.; Gönen, M.; Weng, L.; Green, D.; Erdi, Y.E.; Larson, S.M.; Yeung, H.W. Intensity of^{18}Fluorodeoxyglucose Uptake in Positron Emission Tomography Distinguishes Between Indolent and Aggressive Non-Hodgkin's Lymphoma. *J. Clin. Oncol.* **2005**, *23*, 4643–4651. https://doi.org/10.1200/jco.2005.12.072.
9. Noy, A.; Schöder, H.; Gönen, M.; Weissler, M.; Ertelt, K.; Cohler, C.; Portlock, C.; Hamlin, P.; Yeung, H.W.D. The majority of transformed lymphomas have high standardized uptake values (SUVs) on positron emission tomography (PET) scanning similar to diffuse large B-cell lymphoma (DLBCL). *Ann. Oncol.* **2009**, *20*, 508–512. https://doi.org/10.1093/annonc/mdn657.
10. Shichijo, T.; Maruyama, D.; Yamauchi, N.; Maeshima, A.M.; Sugano, M.; Yuda, S.; Tajima, K.; Kurihara, H.; Shimada, K.; Suzuki, T.; et al. Transformation Scoring System (TSS): A new assessment index for clinical transformation of follicular lymphoma. *Cancer Med.* **2020**, *9*, 8864–8874. https://doi.org/10.1002/cam4.3501.
11. Leonard, J.P.; Trneny, M.; Izutsu, K.; Fowler, N.H.; Hong, X.; Zhu, J.; Zhang, H.; Offner, F.; Scheliga, A.; Nowakowski, G.S.; et al. AUGMENT: A Phase III Study of Lenalidomide Plus Rituximab Versus Placebo Plus Rituximab in Relapsed or Refractory Indolent Lymphoma. *J. Clin. Oncol.* **2019**, *37*, 1188–1199. https://doi.org/10.1200/jco.19.00010.
12. Falchi, L.; Nijland, M.; Huang, H.; Linton, K.M.; Seymour, J.F.; Tao, R.; Kwiatek, M.; Costa, A.; Vassilakopoulos, T.P.; Greil, R.; et al. Epcoritamab, lenalidomide, and rituximab versus lenalidomide and rituximab for relapsed or refractory follicular lymphoma (EPCORE FL-1): A global, open-label, randomised, phase 3 trial. *Lancet* **2025**, *407*, 161–173. https://doi.org/10.1016/s0140-6736(25)02360-8.
13. Sehn, L.H.; Hübel, K.; Luminari, S.; Scholz, C.W.; Salar, A.; Paneesha, S.; Wahlin, B.E.; Panayiotidis, P.; Lee, H.-P.; Jiménez-Ubieto, A.; et al. Tafasitamab, lenalidomide, and rituximab in relapsed or refractory follicular lymphoma (inMIND): A global, phase 3, randomised controlled trial. *Lancet* **2025**, *407*, 133–146. https://doi.org/10.1016/s0140-6736(25)01778-7.
14. Cheson, B.D.; Chua, N.; Mayer, J.; Dueck, G.; Trněný, M.; Bouabdallah, K.; Fowler, N.; Delwail, V.; (Deceased), O.P.; Salles, G.; et al. Overall Survival Benefit in Patients With Rituximab-Refractory Indolent Non-Hodgkin Lymphoma Who Received Obinutuzumab Plus Bendamustine Induction and Obinutuzumab Maintenance in the GADOLIN Study. *J. Clin. Oncol.* **2018**, *36*, 2259–2266. https://doi.org/10.1200/jco.2017.76.3656.
15. Rummel, M.; Kaiser, U.; Balser, C.; Stauch, M.; Brugger, W.; Welslau, M.; Niederle, N.; Losem, C.; Boeck, H.-P.; Weidmann, E.; et al. Bendamustine plus rituximab versus fludarabine plus rituximab for patients with relapsed indolent and mantle-cell lymphomas: A multicentre, randomised, open-label, non-inferiority phase 3 trial. *Lancet Oncol.* **2016**, *17*, 57–66. https://doi.org/10.1016/s1470-2045(15)00447-7.
16. Ladetto, M.; Tavarozzi, R.; Zanni, M.; Evangelista, A.; Ferrero, S.; Tucci, A.; Botto, B.; Bolis, S.; Volpetti, S.; Zilioli, V.; et al. Radioimmunotherapy versus autologous hematopoietic stem cell transplantation in relapsed/refractory follicular lymphoma: A Fondazione Italiana Linfomi multicenter, randomized, phase III trial. *Ann. Oncol.* **2023**, *35*, 118–129. https://doi.org/10.1016/j.annonc.2023.10.095.

17. Schouten, H.C.; Qian, W.; Kvaloy, S.; Porcellini, A.; Hagberg, H.; Johnsen, H.E.; Doorduijn, J.K.; Sydes, M.R.; Kvalheim, G. High-Dose Therapy Improves Progression-Free Survival and Survival in Relapsed Follicular Non-Hodgkin's Lymphoma: Results From the Randomized European CUP Trial. *J. Clin. Oncol.* **2003**, *21*, 3918–3927. https://doi.org/10.1200/jco.2003.10.023.
18. Dreyling, M.; Fowler, N.H.; Dickinson, M.; Martinez-Lopez, J.; Kolstad, A.; Butler, J.; Ghosh, M.; Popplewell, L.; Chavez, J.C.; Bachy, E.; et al. Durable response after tisagenlecleucel in adults with relapsed/refractory follicular lymphoma: ELARA trial update. *Blood* **2024**, *143*, 1713–1725. https://doi.org/10.1182/blood.2023021567.
19. Neelapu, S.S.; Chavez, J.C.; Sehgal, A.R.; Epperla, N.; Ulrickson, M.L.; Bachy, E.; Munshi, P.N.; Casulo, C.; Maloney, D.G.; de Vos, S.; et al. Five-Year Follow-Up Analysis of ZUMA-5: Axicabtagene Ciloleucel in Relapsed/Refractory Indolent Non-Hodgkin Lymphoma. *J. Clin. Oncol.* **2025**, *43*, 3573–3577. https://doi.org/10.1200/jco-25-00668.
20. Morschhauser, F.; Dahiya, S.; Palomba, M.L.; Martin Garcia-Sancho, A.; Reguera Ortega, J.L.; Kuruvilla, J.; Jäger, U.; Cartron, G.; Izutsu, K.; Dreyling, M.; et al. Lisocabtagene maraleucel in follicular lymphoma: The phase 2 TRANSCEND FL study. Nature Medicine. 2024;30:2199-207.
21. Kim, T.; Taszner, M.; Novelli, S.; Cho, S.-G.; Villasboas, J.; Merli, M.; Jiménez-Ubieto, A.; Tessoulin, B.; Poon, L.; Tucker, D.; et al. Safety and efficacy of odronextamab in patients with relapsed or refractory follicular lymphoma. *Ann. Oncol.* **2024**, *35*, 1039–1047. https://doi.org/10.1016/j.annonc.2024.08.2239.
22. Sehn, L.H.; Bartlett, N.L.; Matasar, M.J.; Schuster, S.J.; Assouline, S.E.; Giri, P.; Kuruvilla, J.; Shadman, M.; Cheah, C.Y.; Dietrich, S.; et al. Long-term 3-year follow-up of mosunetuzumab in relapsed or refractory follicular lymphoma after ≥2 prior therapies. *Blood* **2025**, *145*, 708–719. https://doi.org/10.1182/blood.2024025454.
23. Bartlett, N.L.; Sehn, L.H.; Assouline, S.; Giri, P.; Kuruvilla, J.; Schuster, S.J.; Yoon, S.; Fay, K.; Hess, G.; Dreyling, M.; et al. Fixed-Duration Subcutaneous Mosunetuzumab in Relapsed/Refractory Follicular Lymphoma: Pivotal Phase 2 Primary Analysis. *Am. J. Hematol.* **2026**, *101*, 986–997. https://doi.org/10.1002/ajh.70225.
24. Zinzani, P.L.; Mayer, J.; Flowers, C.R.; Bijou, F.; De Oliveira, A.C.; Song, Y.; Zhang, Q.; Merli, M.; Bouabdallah, K.; Ganly, P.; et al. ROSEWOOD: A Phase II Randomized Study of Zanubrutinib Plus Obinutuzumab Versus Obinutuzumab Monotherapy in Patients With Relapsed or Refractory Follicular Lymphoma. *J. Clin. Oncol.* **2023**, *41*, 5107–5117. https://doi.org/10.1200/jco.23.00775.
25. Zinzani, P.L.; Mayer, J.; Flowers, C.; Bijou, F.; De Oliveira, A.C.; Song, Y.; Zhang, Q.-Y.; Brociner, M.; Bouabdallah, K.; Ganly, P.S.; et al. Final analysis of the randomized phase 2 ROSEWOOD study of zanubrutinib + obinutuzumab vs obinutuzumab monotherapy in patients with relapsed/refractory follicular lymphoma. *Blood* **2025**, *146*, 227–227. https://doi.org/10.1182/blood-2025-227.
